# Analytical evaluation of the clonoSEQ Assay for establishing measurable (minimal) residual disease in acute lymphoblastic leukemia, chronic lymphocytic leukemia, and multiple myeloma

**DOI:** 10.1186/s12885-020-07077-9

**Published:** 2020-06-30

**Authors:** Travers Ching, Megan E. Duncan, Tera Newman-Eerkes, Mollie M. E. McWhorter, Jeffrey M. Tracy, Michelle S. Steen, Ryan P. Brown, Srivatsa Venkatasubbarao, Nicholas K. Akers, Marissa Vignali, Martin E. Moorhead, Drew Watson, Ryan O. Emerson, Tobias P. Mann, B. Melina Cimler, Pamela L. Swatkowski, Ilan R. Kirsch, Charles Sang, Harlan S. Robins, Bryan Howie, Anna Sherwood

**Affiliations:** 1 Computational Biology, Adaptive Biotechnologies Corporation, 1551 Eastlake Ave. E, Suite 200, Seattle, WA 98102 USA; 2 Regulatory Affairs, Adaptive Biotechnologies Corporation, 1551 Eastlake Ave. E, Suite 200, Seattle, WA 98102 USA; 3 Research and Development, Adaptive Biotechnologies Corporation, 1551 Eastlake Ave. E, Suite 200, Seattle, WA 98102 USA; 4 Laboratory Operations Improvement, Adaptive Biotechnologies Corporation, 1551 Eastlake Ave. E, Suite 200, Seattle, WA 98102 USA; 5 Molecular Product Development, Adaptive Biotechnologies Corporation, 1551 Eastlake Ave. E, Suite 200, Seattle, WA 98102 USA; 6 Independent Consultant, Adaptive Biotechnologies Corporation, 1551 Eastlake Ave. E, Suite 200, Seattle, WA 98102 USA; 7 Antigen Map, Adaptive Biotechnologies Corporation, 1551 Eastlake Ave. E, Suite 200, Seattle, WA 98102 USA; 8 Software Engineering, Adaptive Biotechnologies Corporation, 1551 Eastlake Ave. E, Suite 200, Seattle, WA 98102 USA; 9 Translational Medicine, Adaptive Biotechnologies Corporation, 1551 Eastlake Ave. E, Suite 200, Seattle, WA 98102 USA; 10 Clinical Diagnostics, Adaptive Biotechnologies Corporation, 1551 Eastlake Ave. E, Suite 200, Seattle, WA 98102 USA; 11 Innovation, Adaptive Biotechnologies Corporation, 1551 Eastlake Ave. E, Suite 200, Seattle, WA 98102 USA

**Keywords:** Analytical validation, Acute lymphoblastic leukemia, Multiple myeloma, Chronic lymphocytic leukemia, Next-generation sequencing, Measurable residual disease, Minimal residual disease, Lymphoma, Leukemia, Myeloma

## Abstract

**Background:**

The clonoSEQ® Assay (Adaptive Biotechnologies Corporation, Seattle, USA) identifies and tracks unique disease-associated immunoglobulin (Ig) sequences by next-generation sequencing of IgH, IgK, and IgL rearrangements and IgH-BCL1/2 translocations in malignant B cells. Here, we describe studies to validate the analytical performance of the assay using patient samples and cell lines.

**Methods:**

Sensitivity and specificity were established by defining the limit of detection (LoD), limit of quantitation (LoQ) and limit of blank (LoB) in genomic DNA (gDNA) from 66 patients with multiple myeloma (MM), acute lymphoblastic leukemia (ALL), or chronic lymphocytic leukemia (CLL), and three cell lines. Healthy donor gDNA was used as a diluent to contrive samples with specific DNA masses and malignant-cell frequencies. Precision was validated using a range of samples contrived from patient gDNA, healthy donor gDNA, and 9 cell lines to generate measurable residual disease (MRD) frequencies spanning clinically relevant thresholds. Linearity was determined using samples contrived from cell line gDNA spiked into healthy gDNA to generate 11 MRD frequencies for each DNA input, then confirmed using clinical samples. Quantitation accuracy was assessed by (1) comparing clonoSEQ and multiparametric flow cytometry (mpFC) measurements of ALL and MM cell lines diluted in healthy mononuclear cells, and (2) analyzing precision study data for bias between clonoSEQ MRD results in diluted gDNA and those expected from mpFC based on original, undiluted samples. Repeatability of nucleotide base calls was assessed via the assay’s ability to recover malignant clonotype sequences across several replicates, process features, and MRD levels.

**Results:**

LoD and LoQ were estimated at 1.903 cells and 2.390 malignant cells, respectively. LoB was zero in healthy donor gDNA. Precision ranged from 18% CV (coefficient of variation) at higher DNA inputs to 68% CV near the LoD. Variance component analysis showed MRD results were robust, with expected laboratory process variations contributing ≤3% CV. Linearity and accuracy were demonstrated for each disease across orders of magnitude of clonal frequencies. Nucleotide sequence error rates were extremely low.

**Conclusions:**

These studies validate the analytical performance of the clonoSEQ Assay and demonstrate its potential as a highly sensitive diagnostic tool for selected lymphoid malignancies.

## Background

The clinical relevance of measurable (minimal) residual disease (MRD) in hematologic malignancies is well established, with increasing evidence supporting the use of MRD as an independent prognostic factor and to guide treatment decisions [1–7]. MRD refers to the number of cancer cells that remain in a person during and following treatment. Recent meta-analyses and an evidence review have shown that, in both adults and children with acute lymphoblastic leukemia (ALL), event-free survival (EFS), relapse-free survival (RFS), and overall survival (OS) are significantly associated with MRD levels measured at the end of induction treatment [1, 2, 5]. Similar findings have been reported in meta-analyses of studies in patients with multiple myeloma (MM) [8] and in those with chronic lymphocytic leukemia (CLL) [9].

MRD monitoring to inform patient outcomes and treatment choice is discussed in clinical practice guidelines for several indications [4, 10–18]. The widespread adoption of MRD monitoring in everyday clinical practice will depend upon the availability of accurate and reliable assays to measure and track disease burden over time. Many institutions currently measure MRD using multiparametric flow cytometry (mpFC); this method is relatively fast and provides information at a cellular level, but is limited by problems with standardization and reproducibility [19, 20]. Allele-specific oligonucleotide real-time quantitative polymerase chain reaction (ASO-PCR) is a sensitive alternative for detecting MRD, but is time-consuming and difficult to standardize because it depends on the development of patient-specific primers [19, 20]. Next-generation sequencing (NGS) offers an alternative approach that is reproducible, highly sensitive, and does not require patient-specific primers, which allows reliable identification and quantitation of unique immunoglobulin (Ig) rearrangements in hematologic malignancies.

**Fig. 1:**
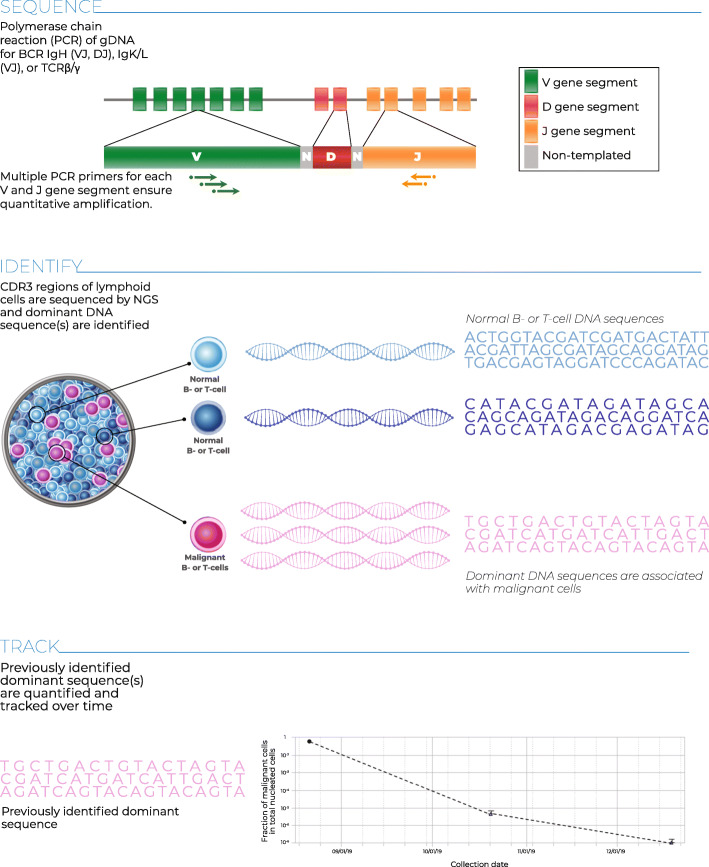
The clonoSEQ Assay Processg: DNA is extracted from the patient sample, and the CDR3 regions of B- and T-cell receptors are subject to multiplexPCR to amplify their unique VDJ or VJ sequences. Amplified DNA undergoes a second round of PCR to add index sequences to prepare for NGS, which is performed via synthesis. The resulting sequences are processed by bioinformatics software to ensure accuracy of results

The clonoSEQ® Assay (Adaptive Biotechnologies; Seattle, WA) is an in vitro diagnostic (IVD) test that uses multiplex PCR and NGS to identify and quantify disease-associated sequence rearrangements (or clonotypes) of the IgH, IgK, and IgL receptor genes, as well as IgH/BCL1 and IgH/BCL2 translocations, in DNA extracted from bone marrow [21, 22]. The Assay has been FDA cleared for assessing MRD in bone marrow samples in MM and ALL. clonoSEQ is also available for use in other B and T cell malignancies as a laboratory developed test (LDT). Once disease-associated clonotypes have been identified in a diagnostic (or ‘ID’) sample from a patient, the assay can be used to detect the level of residual disease in follow-up samples (‘MRD’ samples) from the same patient by tracking the presence and frequency of these clonotypes (Fig. 1).

Here, we present the results of studies designed to validate the analytical performance of the clonoSEQ Assay using clinical bone marrow samples and cell lines from 3 disease conditions: ALL, CLL, and MM.

## Methods

All of the studies described used prespecified standard operating procedures, statistical analysis plans, and acceptance criteria, as well as using qualified critical reagents, instruments and software, and traceable reagent lots. Study designs followed established Clinical and Laboratory Standards Institute (CLSI) guidelines when relevant [23–26].

### Sample selection

Clinical samples were obtained from clinical collaborators and commercial vendors, for a total of 115 patients diagnosed with MM, ALL, or CLL [samples were derived from bone marrow aspirate (BMA) and peripheral blood]. All clinical disease samples had been previously characterized by mpFC and/or immunohistochemistry to independently quantify disease burden. In addition, cell lines for each lymphoid malignancy were purchased; these comprised MM lines IM-9 (ATCC; Manassas, VA), L-363 (Leibniz Institute DSMZ; Germany), NCI-H929 (Sigma; St. Louis, MO), and U-266 (ATCC); ALL lines GM14952 (Coriell; Camden, NJ), GM20390 (Coriell), and SUP-B15 (ATCC); and CLL lines MEC-1 (DSMZ), HG-3 (DSMZ), and PGA-1 (DSMZ). Genomic DNA (gDNA) was extracted using an automated QIAsymphony SP® instrument (QIAGEN; Hilden, Germany) and the gDNA concentration was measured by the Quant-iT™ PicoGreen® assay (Thermo Fisher Scientific; Waltham, MA). A subset of 66 clinical samples (21 ALL, 22 CLL, and 23 MM samples) was chosen for use in these analytical validation studies; samples were preferentially selected to have high disease burdens and high mass of gDNA since the contrived samples generated for these studies required higher volumes and tumor burdens than samples submitted for routine clinical assessment. Samples were also selected to provide representative proportions of non-unique clonotype sequences (relative to previously assayed clinical samples) while ensuring that no two samples carried an identical clonal sequence. Contrived samples were prepared by mixing gDNA from these 66 clinical samples and 9 cancer cell lines with gDNA from the bone marrow of 7 healthy subjects (Table S1).

### MRD detection and tracking by the clonoSEQ assay

Cancer clonotype sequences are identified in diagnostic ‘ID’ samples and then measured in follow-up MRD samples using the clonoSEQ Assay. Genomic DNA is amplified using locus-specific multiplex PCR with a master mix of primers targeting V, D, and J genes of the IgH, IgK, IgL, BCL1/IgH and BCL2/IgH loci; a second PCR is used to add reaction-specific barcodes for sample identification. The assay also amplifies genomic regions present as diploid copies in normal gDNA to quantify the total nucleated cell content of a sample. Barcoded amplicons are then pooled into sequencing libraries, checked for adequate DNA amplification by quantitative PCR (qPCR), and sequenced using the Illumina NextSeq™ 500 System (Illumina; San Diego, CA). The target mass of input DNA for ID samples is 500 ng and for MRD samples, 20 μg; in practice, MRD samples may contain more or less DNA than the targeted amount, so this study includes samples with < 500 ng to 40 μg of DNA to capture the full range of acceptable inputs to the assay. Positive and negative amplification and sequencing controls are included in each reaction batch to ensure that all steps meet predefined quality thresholds.

Sequencing data are processed using a custom bioinformatics pipeline, with data quality checked at the flowcell, PCR well, and sample levels. Reads are assigned to rearranged B-cell receptors (BCRs) for each sample and clustered into clonal receptor sequences; these sequences are assessed for their likelihood to be disease associated and their suitability for subsequent tracking. A sequence is considered acceptable for tracking if it comprises at least 3% of all BCR sequences at a given locus and at least 0.2% of all nucleated cells in the sample, is well separated from the background repertoire (no more than 5 other less-abundant sequences from the same locus with repertoire frequencies within a factor of 10 of the frequency of the sequence selected for tracking), is represented by at least 40 gDNA templates, and is sufficiently unique for tracking. Sequence uniqueness is assessed by comparison with a large database of previously observed Ig rearrangements; depending on its incidence in the database, each sequence is assigned a uniqueness score that reflects its likelihood of being detected in a healthy repertoire. Sequences with poor uniqueness scores are excluded from MRD tracking; this prevents false MRD signals from being generated by healthy clones with Ig rearrangements that coincidentally match sequences from a malignant clone.

Once suitable disease-associated sequences have been identified, these ID sequences are compared with those found in successive MRD sample(s) for tracking. Imperfect matching between ID and MRD sample sequences is permitted to account for potential somatic mutations in a disease-associated sequence; sequences with higher complexity (hence lower probability of independently forming in a non-malignant clonal population) are permitted to include a higher proportion of mismatched nucleotides. Finally, the abundance of each of the tracked sequences in an MRD sample is measured and used to compute a consensus sample-level malignant cell count and a total nucleated cell count. The ratio of these values provides an estimate of the MRD frequency in a sample.

### Sensitivity and specificity

The goal of this analysis was to determine the sensitivity and specificity of the clonoSEQ Assay by assessing the limit of detection (LoD), the limit of quantitation (LoQ) and the limit of blank (LoB). These parameters were required in order to make sample-level MRD estimates for the subsequent evaluation studies.

The LoD was defined as the malignant-cell count at which the assay would detect MRD in 95% of samples. The LoQ was defined as the lowest clonoSEQ sample MRD frequency that could be quantitatively determined within 70% relative total error, defined as root-mean-square error (RMSE) divided by the number of input malignant cells. RMSE can be calculated as the square root of the squared bias plus the variance. An allowable 70% total error near the LOD of the assay is acceptable for the intended clinical use of the assay. At this level of total error, if two malignant cells were truly present in a sample (which is near the expected LOD), 95% of MRD measurements would report between 1 and 5 malignant cells. This would not significantly change the interpretation of the MRD result.

The LoD and LoQ of the clonoSEQ Assay were estimated and confirmed in 2 sequential experiments. gDNA from 66 clinical disease samples and 3 cell lines (1 for each lymphoid malignancy: GM14952, IM-9, MEC-1) was pooled at specific ratios according to the sample disease loads, such that each sample contained the same expected number of malignant cell equivalents. gDNA from 7 healthy donors was also pooled. The healthy gDNA pool was then used as a diluent for the disease gDNA pool to generate contrived samples with specific DNA masses and malignant cell frequencies.

The first experiment estimated the LoD and LoQ using DNA input amounts of 500 ng and 20 μg, each using 5 MRD frequencies ranging from 1 to 23 malignant cells per disease sample. This experiment generated LoD and LoQ estimates based on the combined data from all 3 disease indications and both DNA input amounts. The second experiment was designed to confirm the estimated LoD and LoQ using 8 input DNA concentrations across the entire input range from 500 ng to 20 μg. DNA input levels above and below the range (40 μg and 200 ng, respectively) were also included. For each input DNA concentration, the MRD frequencies estimated in the first experiment (in units of ‘malignant cell equivalents,’ which are independent of DNA input amount) were tested. In both the first and second experiments, each of the contrived samples was tested in duplicate with the clonoSEQ Assay using 1 operator set, 1 instrument set, and 4 reagent lots.

The LoB was determined by assessing the presence and abundance of a patient’s trackable malignant Ig sequences, as defined by the corresponding MRD frequencies, in healthy bone marrow. The MRD frequency that would be observed by chance in up to 5% of healthy repertoires, assuming a given amount of available gDNA, was then identified. This metric reflects the probability that a non-malignant clone would independently rearrange the same Ig receptor sequence as a malignant clone and not be excluded by the tracking algorithm, which could lead to an inflated MRD abundance estimate or false detection of MRD. While the LoB was defined in this study to control for a type I error rate of 5%, it was expected that the true false detection rate of the assay would be much less than 5% since the majority of sequences selected for MRD tracking are highly specific to the malignant clone from a given patient. During sample preparation, the calibrated clonotype sequences had all been identified as independent, and therefore none were excluded from this analysis.

Trackable malignant Ig sequences identified in the 66 patient samples were searched for in bone marrow-derived gDNA from 7 healthy donors at 3 DNA input amounts, 500 ng, 20 μg and 40 μg, respectively, which correspond to the minimum, target, and maximum range of the clonoSEQ Assay for MRD samples. Each of these 21 samples was tested with the clonoSEQ Assay using 1 operator set and 1 instrument set. At least 2 reagent lots were used for all test samples (4 reagent lots were used for the 500 ng and 20 μg samples, and 2 were used for the 40 μg sample). For each DNA input, 28 samples (7 × 4) were used to assess LOB.

#### Statistical analysis

To determine the LoD, the proportion of MRD positive results obtained from the clonoSEQ Assay was modeled as a function of expected clonal frequency (based on disease loads estimated by the clonoSEQ Assay in the undiluted samples, plus subsequent dilution factors) using a probit model. The LoD was calculated as the expected number of malignant input cells at which the fitted probit curve reached a detection probability of 95%.

The LoQ was estimated using Sadler’s precision profile model to relate expected clonal frequencies to relative total error estimates [27]. Sadler’s precision profile model is a flexible three-parameter model for regressing variance as a function of input. The form of the model is:

$$ y={\left({\beta}_1+{\beta}_2x\right)}^J $$

Here *β*_1_, *β*_2_ and *J* are free parameters which convert the input, *x*, into an estimate of the variance or total error, *y*. The LoQ was calculated as the expected number of malignant input cells at which the fitted precision profile curve reached a relative total error of 70%.

The LoB was estimated in the 20 μg samples (which are most likely to contain sequences from non-malignant clones which match a tracked sequence) and confirmed in the 500 ng samples. Non-parametric statistics were used to find the 95th percentile of MRD measurements among all tracked sequences in all blank samples at each DNA input level. These analyses were independently repeated in the 40 μg samples to confirm LoB.

### Precision

#### Study design

**Fig. 2:**
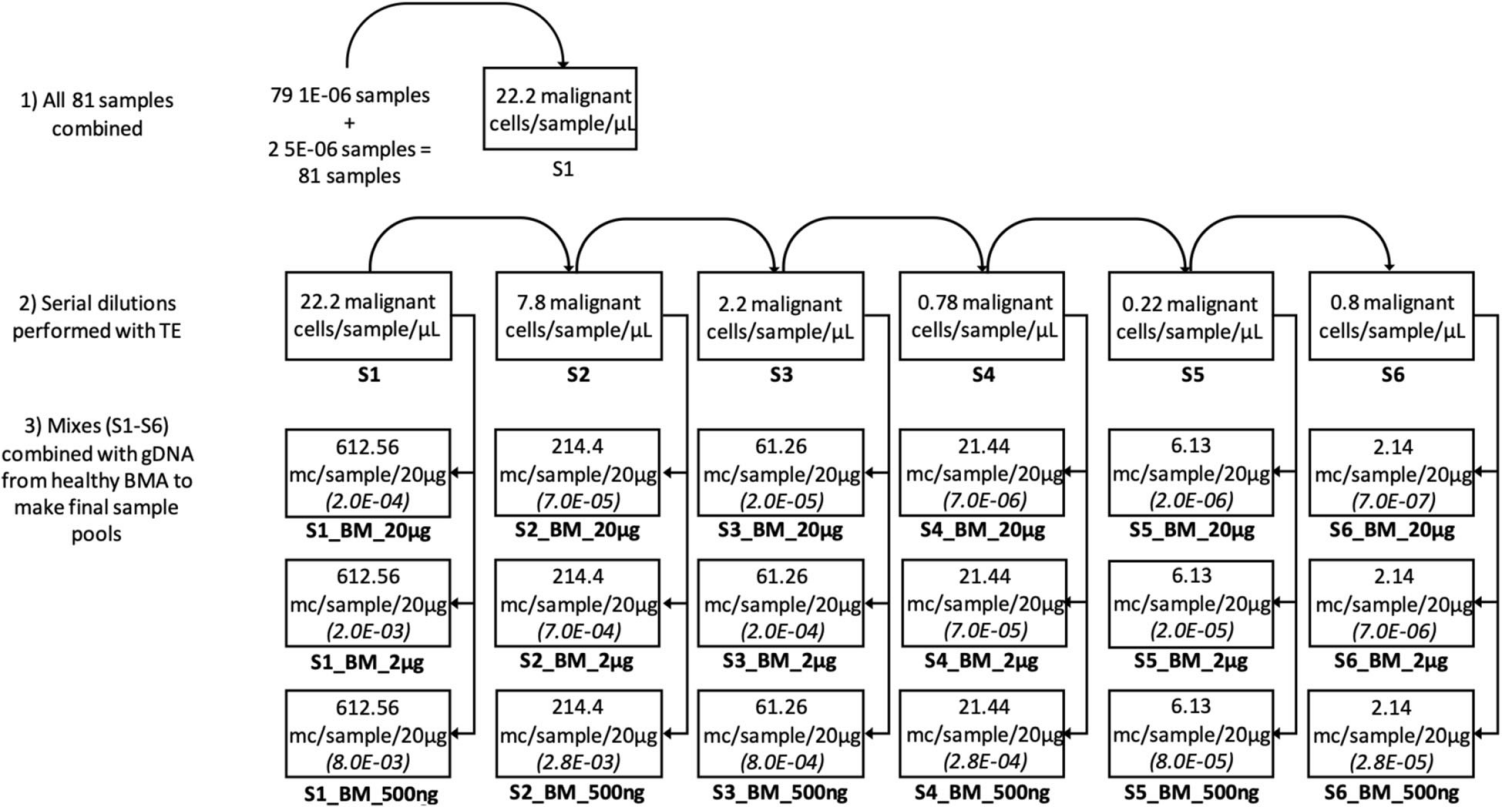
Preparation of total gDNA input samples for precision analysis and MRD frequencies used in Linearity testing. Frequencies are presented parenthetically; sample names are presented below the boxes; pre-dilution malignant cell concentrations were determined by mpFC and/or immunohistochemistry. Abbreviations for image: BM bone marrow, BMA bone marrow aspirate, gDNA genomic DNA, mc malignant cells, OPA overall percent agreement

The primary goal of this study was to analytically validate the precision of the clonoSEQ Assay using clinical samples from 3 indications (MM, CLL, and ALL). Contrived disease samples were generated by diluting gDNA combined from 66 patient clinical samples with gDNA pooled from BMA from 7 healthy donors, to achieve 6 malignant cell frequencies in total DNA input amounts of 500 ng, 2 μg, and 20 μg (Fig. 2).

The precision, repeatability and reproducibility study used a main effects screening design over 21 calendar days and 10 assay runs to measure the effects of day, run within day, operator set (3 sets), instrument set (2 sets of thermal cycler/liquid handler matrixed with 2 sequencers), and reagent lot (4 lots) for each disease indication and sample MRD frequency under study (Fig. S1). The disease-associated sequences from each clinical sample which were identified during ID testing were searched for in all contrived samples, generating a sample MRD frequency measurement for each of the 66 clinical samples in each contrived sample. These sample MRD measurements were then used to determine the precision of the clonoSEQ Assay.

#### Statistical analysis

For each DNA input level and sample MRD frequency measurement, mixed models and analysis of variance (ANOVA) were used to model MRD measurements as a function of different operator sets, instrument sets, reagent lots, days, and runs within day, while treating each variable as a random effect. This information was used to decompose the total variability in MRD measurements for each input DNA level into components of variance attributable to each variable and to random error. All data points with expected MRD levels below the LoD of a sample were excluded from analysis.

Estimates of repeatability were obtained from the component of variance associated with random error, which included the variability associated with duplicate measurements under the same experimental conditions. Estimates of reproducibility were obtained from the sum of the components of variance due to operator set, instrument set, reagent lot, day, run within day, and random error; estimates of lot-to-lot variability were obtained from the component of variance associated with reagent lot. The percentage coefficient of variation (%CV) due to repeatability, reproducibility, and lot-to-lot variability in replicated MRD measurements was then calculated for each input DNA level and targeted sample MRD frequency.

### Linearity

#### Study design

The primary goal of this analysis was to analytically validate the linear range of the clonoSEQ Assay. Contrived disease samples across a range of malignant cell frequencies were created by spiking gDNA from the 9 cell lines (3 for each of MM, CLL, and ALL, as detailed above; only MM and ALL for the 40 μg DNA input) into background gDNA pooled from the whole blood of 3 healthy donors. Four DNA input amounts (200 ng, 2 μg, 20 μg, and 40 μg) were tested, which cover the acceptable range of inputs for MRD testing (500 ng–40 μg). While the minimum input for MRD testing via the clonoSEQ Assay is 500 ng (to ensure sensitivity at an MRD frequency of 1 × 10^− 4^), we included a 200 ng input level to assess whether linearity extends beyond the range of the currently acceptable MRD testing input, as well as a 40 μg input level to measure linearity beyond the targeted MRD input of 20 μg. Genomic DNA from cancer cells was spiked into the background gDNA at frequencies ranging from just below the expected LoQ of 2.5 cancer cells to hundreds of thousands of cancer cells comprising up to 100% of nucleated cells in a sample (Table 1). The frequencies estimated by the assay were then checked for linearity across clinically relevant ranges for MRD testing.

**Table 1:** Disease-associated clone frequency ratios assessed in linearity study

			Total DNA Input			
			40 μg	20 μg	2 μg	200 ng
Replicates		# of cancer cells	6,125,574	3,062,787	306,279	30,628
8	Freq 1	2		0.000065%	0.00065%	0.0065%
8	Freq 2	2.5		0.000082%	0.00082%	0.0082%
8	Freq 3	3		0.0001%	0.001%	0.01%
4	Freq 4	4.6	0.000075%			
3	Freq 5	6.1	0.0001%			
4	Freq 6	9.2		0.0003%	0.003%	0.03%
2	Freq 7	30.6		0.001%	0.01%	0.1%
4	Freq 8	61.3	0.001%			
2	Freq 9	91.9		0.003%	0.03%	0.30%
2	Freq 10	306.3		0.01%	0.10%	1%
3	Freq 11	612.6	0.01%			
2	Freq 12	918.8		0.03%	0.30%	3%
2	Freq 13	3062.8		0.1%	1%	10%
4	Freq 14	6125.6	0.1%			
2	Freq 15	9188.4		0.30%	3%	30%^a^
2	Freq 16	30,627.9		1%	10%	100%^a^
4	Freq 17	61,255.7	1%			
2	Freq 18	91,883.6		3%	30%^a^	
2	Freq 19	306,278.7		10%^b^	100%^a^	
4	Freq 20	612,557.4	10%^b^			

*Freq* frequency

1 human diploid cell = 6.53 pg

^a^Single cell line in test, not mixed with other cell lines

^b^3 Cell lines for each cancer type were combined; then CLL, MM, and ALL were tested separately

Assay linearity was confirmed using data from the precision study, in which clinical sample gDNA was diluted with gDNA from pooled healthy individuals. Three representative clinical samples from each disease indication (totaling 9 samples) from the precision study were selected. Linearity assessment was conducted across 6 MRD frequencies at each DNA input: 500 ng, 2 μg, and 20 μg. The range of MRD frequencies tested for each DNA input amount is shown in Fig. 2.

#### Statistical analysis

Linearity was assessed by comparing the proportionality of individual MRD measurements to expected clone frequencies using the polynomial method [28]. First, the data in the verification range were fitted to regression models with first-order (linear), second-order (quadratic), and third-order (cubic) polynomials. If none of the non-linear terms in the second- and third-order polynomials were significant at *P* < 0.05, linearity was established across the verification range. Otherwise, the higher-order polynomial model with the best fit was compared to the linear model at each clonal frequency. If the fitted polynomial was within ±5% of the linear fit at every frequency, the results were considered acceptably linear; otherwise, the range of clonal frequencies was reduced and this procedure repeated until linearity was achieved.

### Quantitation accuracy

#### Study design

The primary aim of these studies was to assess the analytical quantitation accuracy (or bias) of the clonoSEQ Assay relative to mpFC. Two types of experiment were conducted for this purpose: first, 2 ALL cell lines (SUP-B15, GM20390) and 2 MM cell lines (NCI-H929, U266) selected by the mpFC lab were diluted into healthy background mononuclear cells at 5 dilution levels from 5 × 10^− 7^ to 1 × 10^− 2^, with 2 replicates per sample. Second, the data generated in the precision study were re-analyzed for quantitation bias between clonoSEQ MRD measurements in diluted gDNA samples and expected MRD levels based on mpFC measurements of the original gDNA samples and subsequent dilution factors. The Pearson R^2^ coefficient was calculated to assess correlation.

#### Statistical analysis

For the study of cell lines blended with background mononuclear cells, MRD frequencies between mpFC and the clonoSEQ Assay were compared to demonstrate concordance.

For the re-analysis of data from the precision study, which provided a much larger number of data points, a nested bootstrap procedure incorporating random sampling with replacement from hierarchical correlated data was used to account for dependencies among samples and replicate measurements; bootstrap sampling was done separately for each disease indication and number of input cancer cells. Estimated clonoSEQ Assay bias was presented as relative bias (i.e., the difference between observed and expected over expected), along with non-parametric 95% confidence intervals (CI) determined by 10,000 bootstrap replicates. We anticipated a (relative) mean bias of ±35%, which is small relative to clinically meaningful changes in MRD level, and that this bias would remain within ±35% across the tested range of disease burden.

### Sequence accuracy

#### Study design

This study assessed the observed rate of agreement between the nucleotide sequences identified in ID samples for tracking during sample selection and the nu2’cleotide sequences identified in the contrived samples used in the precision study, both as described above.

#### Statistical analysis

For each clonotype sequence designated for tracking, all sequences in an MRD sample within Hamming distance ≤ *N* bp were included for assessment of overall percent agreement (OPA), where *N* was defined for each tracked sequence as the number of allowable mutations based on the complexity (or uniqueness) of the clonotype rearrangement. *N* was chosen to capture somatic genetic variation among B cells from the same clonal lineage without incorrectly grouping sequences from different clonal lineages. Once this population was established, the OPA between the original clonotype sequence and the sequences identified in the MRD assessment was calculated. All OPA values were also restated as a Phred quality score [i.e., −log_10_ (disagreement rate)].

The following algorithm was used to assess OPA:

Given:

- Length (of alignment between MRD sequence and tracked ID clonotype)
- Mismatches (number of mismatched bases in alignment)
- Allowed (allowed mutations for the tracked ID clonotype)
- Abundance (estimated number of templates for MRD sequence)

If (Mismatches ≤ Allowed):

- Positive Agreement = (Length - Mismatches)*Abundance
- Negative Agreement = Mismatches*Abundance

Across all sequences with (Mismatches ≤ Allowed):

- OPA = 100*sum (Positive Agreement)/[sum (Positive Agreement) + sum (Negative Agreement)]

This algorithm measures the degree of nucleotide agreement for each malignant clonotype in complex mixed samples, conditional on certainty (through the number of allowed mutations) that the sequence is genuinely a derivative of the malignant clonotype sequence and not a chance rearrangement within a separate clonal population.

## Results

### Sensitivity and specificity

#### Limit of detection and limit of quantitation

Based on the combined data from ALL, CLL, and MM samples across 2 DNA input levels (500 ng and 20 μg), a probit approach was used to estimate the LoD to be 1.903 malignant cells (95% CI; 1.75–2.07) (Fig. S2; Table 2). This corresponds to a minimal disease burden of 6.77 × 10^− 7^ (6.02 × 10^− 7^–7.61 × 10^− 7^) cells, at an input level of 20 μg of DNA. For samples with MRD below this level, non-detection is more likely to represent an absence of gDNA templates going into the assay (due to subsampling of the gDNA pool) than a technical failure, as explained in the Discussion.

**Table 2:** LoD and LoQ of the clonoSEQ Assay by MRD cell counts and MRD frequency

Measure	Malignant cells^a^ (95% CI)	500 ng DNA input frequency (95% CI)	20 μg DNA input frequency (95% CI)
LoD	1.903 (1.75–2.07)	2.26 × 10^− 5^ (2.01 × 10^− 5^–2.53 × 10^− 5^)	6.77 × 10^− 7^ (6.02 × 10^− 7^–7.61 × 10^− 7^)
LoQ	2.390 (1.90–9.14)	2.39 × 10^−5^ (2.26 × 10^− 5^–7.01 × 10^− 5^)	1.76 × 10^−6^ (6.77 × 10^−7^–4.09 × 10^− 6^)

*CI* confidence interval, *LoD* limit of detection, *LoQ* limit of quantitation, *MRD* minimal residual disease

^a^Calculated from samples with 500 ng and 20 μg of DNA input

Using the same data set, the LoQ was determined to be 2.390 malignant cells (95% CI: 1.903–9.137) (Table 2). Both the LoD and LoQ values correspond to different MRD frequencies at the 2 different cellular inputs since the denominator is different (Table 2). Having an LoQ that is only slightly higher than the LoD confirms that the assay can accurately and precisely quantify gDNA templates even at very low abundance.

Follow-up studies confirmed the LoD and LoQ across total DNA inputs ranging from 200 ng to 40 μg (Fig. S3). The results verified that the LoD and LoQ are consistent (when expressed in units of malignant cells) across a wide range of DNA input levels, thus highlighting the ability of the clonoSEQ Assay to detect and quantify malignant gDNA templates at low levels in any sample.

#### Limit of blank

The LoB of the assay was found to be zero at both the 500 ng and the 20 μg gDNA input levels, confirming that < 5% of MRD measurements in healthy samples produced non-zero values. As anticipated, the false detection rate of MRD in these samples was actually less than 1%, and no MRD estimate was higher than 3 templates. Non-zero MRD measurements in non-malignant cell populations typically represent receptors with intermediate sequence complexity; they are not completely unique to a given patient, but they occur at a low enough rate in the population that they are still useful for MRD tracking. The implications of tracking these kinds of sequences are considered further in the Discussion.

### Precision

Using a mixed-effects model to assess sources of variability, we calculated precision estimates (as % CV) by MRD abundance for each component of variance across the combined DNA input levels (500 ng, 2 μg, and 20 μg) and disease indications (MM, CLL, and ALL) (Table 3).

**Table 3:** Summary of the clonoSEQ Assay precision

		%CV attributed to each variable at cell inputs^a^					
Lot-to-lot variability	Number of input cancer cells	2.14	6.13	21.44	61.26	214.4	612.56
	Instrument set (%)	0	1	0	1	1	1
	Operator (%)	2	0	1	2	0	0
	Processing day (%)	0	0	1	1	0	3
	Processing run (%)	0	0	1	0	0	0
	Reagent lot (%)	0	0	0	1	2	1
	Residual variability (%)	68	49	28	23	19	18
	Total MRD measurements, *n*	3456	3456	3564	3960	3960	3828

*%CV* percent coefficient of variance, *ALL* acute lymphoblastic leukemia, *CLL* chronic lymphocytic leukemia, *MM* multiple myeloma

^a^These values were aggregated across diseases (ALL, CLL, and MM) and total DNA input levels

Precision was primarily influenced by the number of cells being evaluated, and ranged from 68% CV at the lowest spike-in level of 2.14 cancer cells to 18% CV at a spike-in level of 612.56 cells. Notably, measurements at the low end of the MRD range (near the LoD) showed nearly the best possible precision given Poisson variation among contrived samples; e.g., for a diluted sample with an expectation of 2 malignant input cells, even a perfect assay could not achieve less than ~ 70% CV because each dilution series produces stochastic variation around the targeted number of templates. In addition, measurements from the assay were robust to typical variation in lab process features: most of the observed variation in MRD estimates was due to residual variability, with the tested process features (including operator set, instrument set, reagent lot, day, and run within day) contributing only 0 to 3% CV. Precision estimates by disease indication at each input gDNA level are provided in Tables S2, S3 and S4.

**Fig. 3:**
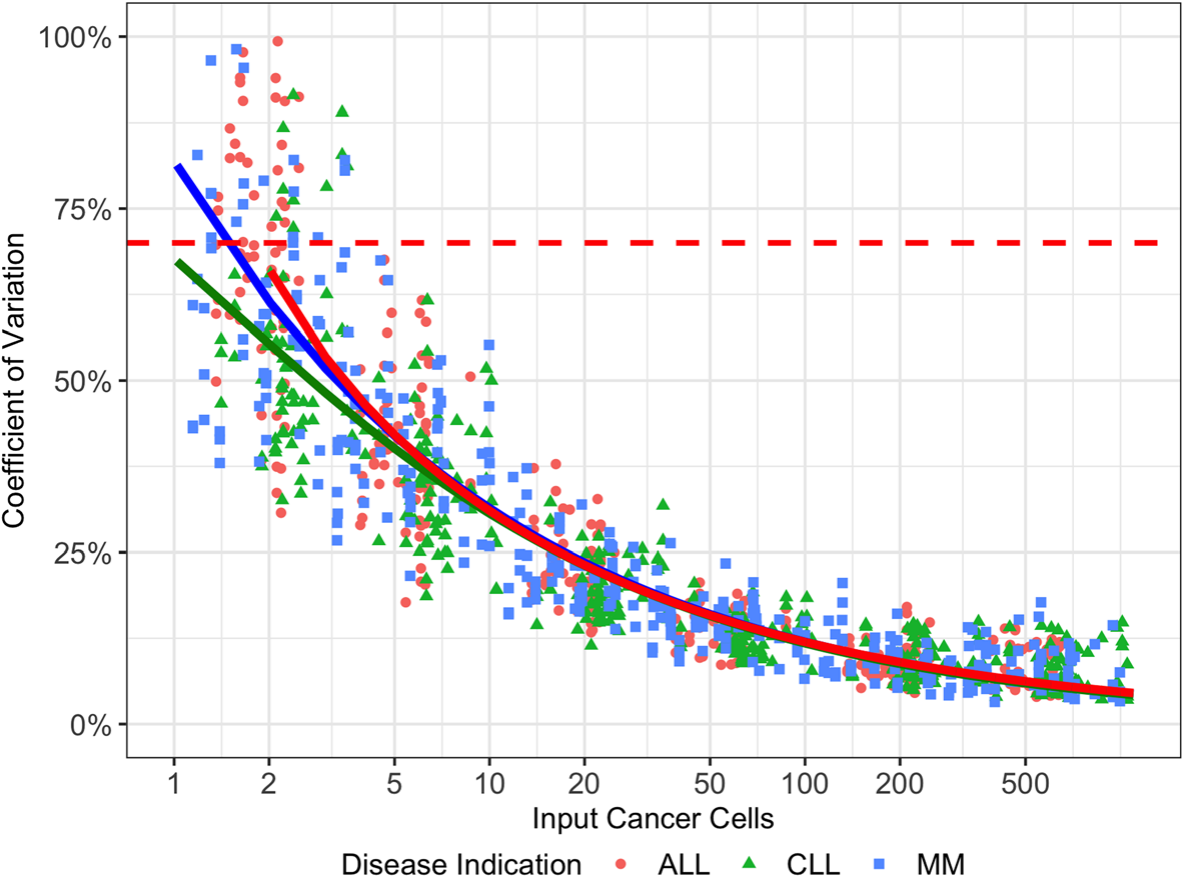
Precision of the clonoSEQ Assay as a function of input cancer cellsThe red dashed line is at 70%, which is the total error threshold used to define the LOQ of the clonoSEQ Assay.Abbreviations for image: ALL acute lymphoblastic leukemia, CLL chronic lymphocytic leukemia, MM multiple myeloma

As summarized in a Sadler’s precision profile, precision of the clonoSEQ Assay was similar for each indication evaluated (Fig. 3). These profiles showed that imprecision (measured by %CV) decreased as more malignant cells were sampled. The data in Fig. 3 were aggregated across disparate gDNA input levels (500 ng, 2 μg, and 20 μg), and the clear trends confirm that the precision of the assay is mainly driven by the number of malignant cells being evaluated while being independent of the total amount of input DNA. This finding illustrates the value of providing large amounts of input gDNA to the assay: for a given MRD frequency, samples with more input DNA will include more copies of the malignant clone, leading to increased precision in quantifying MRD (as well as increased sensitivity).

### Linearity

**Fig. 4:**
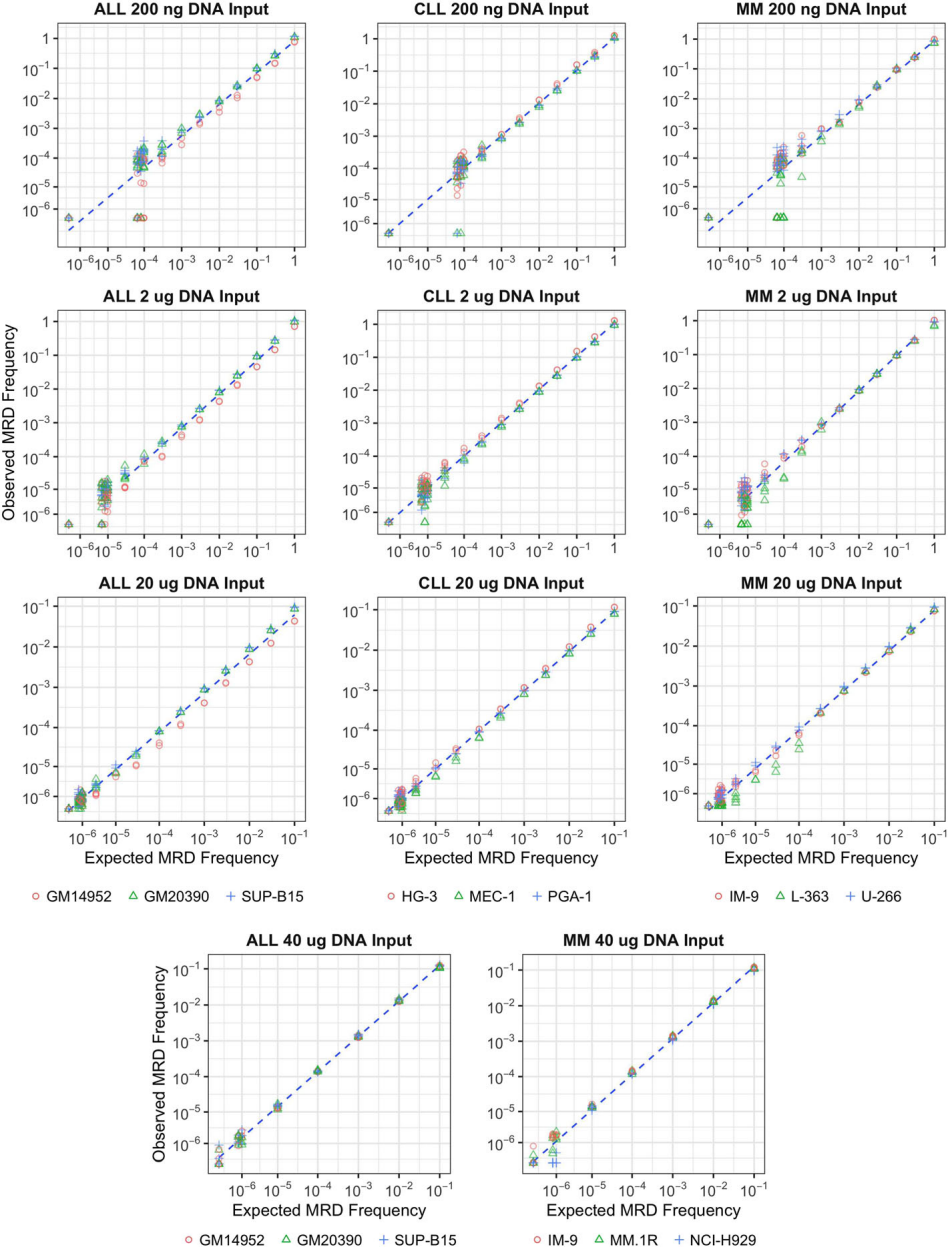
Linearity plots for the clonoSEQ Assay by gDNA input level and disease(ALL, CLL, and MM) Abbreviations for image: ALL acute lymphoblastic leukemia, CLL chronic lymphocytic leukemia, MM multiple myeloma, MRD minimal residual disease

From the results of tests using cell lines, linearity was established over several orders of magnitude across the entire range tested for the 200 ng, 2 μg, and 20 μg sample inputs over all disease indications (ALL, CLL, and MM) and for the 40 μg sample input in MM and ALL (Fig. 4). For gDNA levels from 200 ng to 40 μg (which go beyond the acceptable range of MRD inputs for the assay), estimated slopes for each disease varied from 0.95–1.03, indicating strong proportionality between observed and expected clonal frequencies (Table S5).

Linearity was subsequently confirmed across a range of MRD frequencies using clinical sample data from the precision study (Table S6).

### Accuracy

#### Quantitation accuracy

**Fig. 5:**
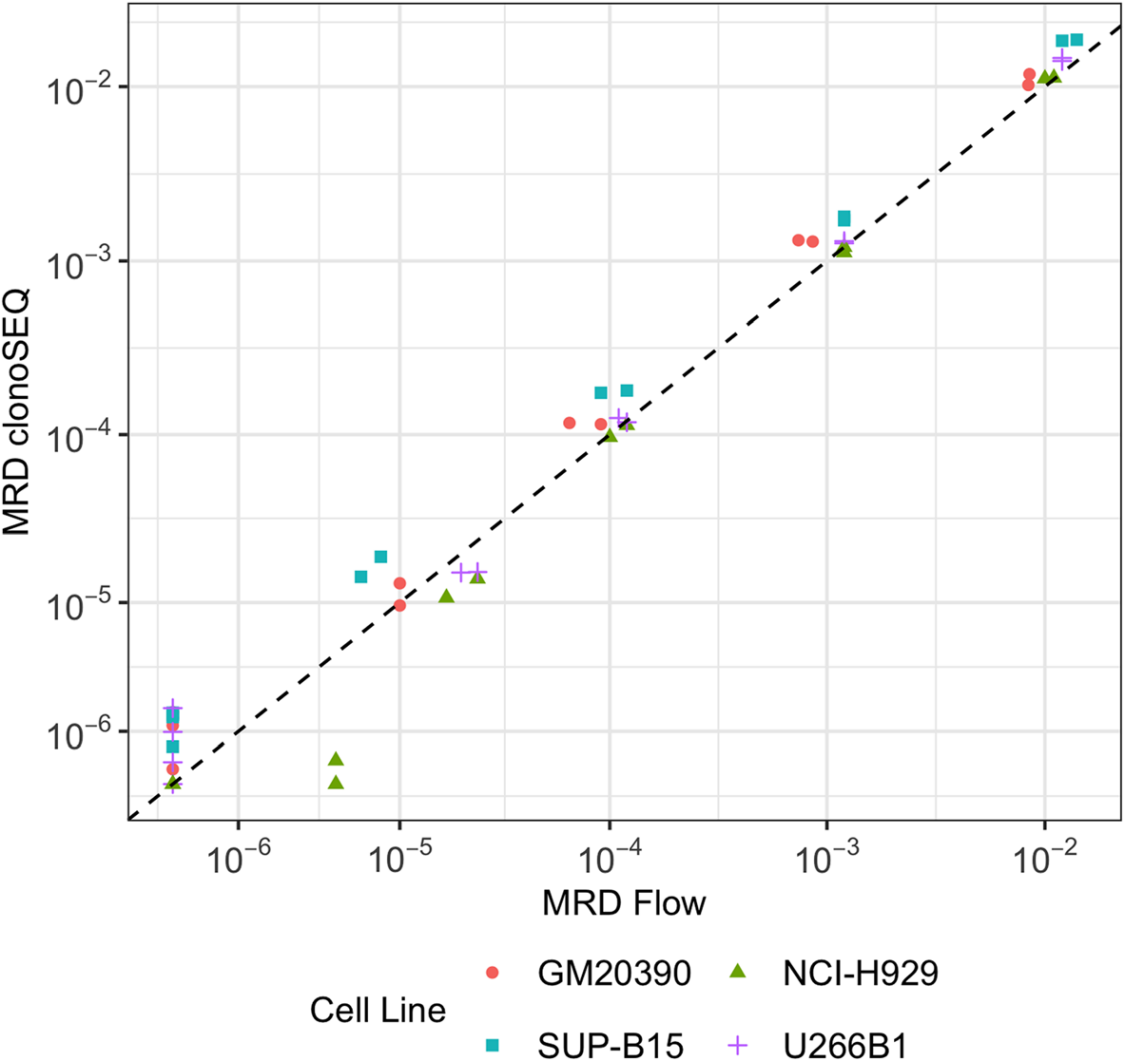
Pairwise comparison of MRD frequency measurements from multiparametric flow cytometry (mpFC; x-axis) and the clonoSEQ Assay (y-axis) for ALL and MM. R = 0.98 Abbreviations for image: Flow, mpFC.

A direct pairwise comparison of quantitative accuracy between the clonoSEQ Assay and mpFC using 2 ALL and 2 MM cell lines showed similar quantitative accuracy across the tested range, particularly at MRD frequencies above 10^− 4^ (Fig. 5). The Pearson R^2^ value was 0.98.

The quantitation accuracy of the clonoSEQ Assay was also assessed in clinical samples by comparison to expected MRD frequencies from mpFC measurements and prescribed dilution factors. For reference, a comparison between disease burden estimated by the clonoSEQ Assay and mpFC in the pre-dilution samples is shown in Figure S4. This analysis showed that the quantitation accuracy was within ±25% across all tested disease cell inputs for ALL and MM (Fig. S5); CLL showed a similar trend, but with an upward shift in the measured bias. Overall, relative bias between disease burden tended to increase at lower cell inputs, a test range that spans the clonoSEQ Assay’s LOQ (2.390 cells). These data show that mpFC and the clonoSEQ Assay report similar disease burdens. The clonoSEQ Assay maintains accurate reporting of disease burden down to ~ 2 input cells in 3 million total cells.

#### Sequence accuracy

The test for sequence accuracy assessed approximately 442.5 million nucleotides for sequence agreement between the original calibrating clonotype sequence (ID sample) and the sequences identified in the MRD assessment. The overall observed sequence error rate was approximately 3.5 parts per 100,000 (Table 4), corresponding to a Phred score of approximately 44.5.

**Table 4:** Summary of sequence agreement metrics

Number of allowed mutations	Nucleotides assessed	OPA	95% CI		Phred^a^
			Lower limit	Upper limit	
1	135,025,044	99.9968	99.9967	99.9969	44.9
2	57,248,770	99.9965	99.9964	99.9967	44.6
3	151,018,837	99.9965	99.9965	99.9966	44.6
4	82,780,612	99.9960	99.9959	99.9962	44.0
5	13,918,166	99.9966	99.9963	99.9969	44.6
6	2,587,014	99.9961	99.9953	99.9968	44.1

*CI* confidence interval, *OPA* overall percent agreement

^a^Phred defined as -log_10_(disagreement rate)

## Discussion

The use of MRD assessment and monitoring as a tool for predicting patient outcomes and informing treatment is now standard clinical practice for adult and pediatric patients with ALL [29]. It is required by the latest International Myeloma Working Group response criteria [13], is increasingly incorporated into follow-up after stem cell transplant in patients with MM [30, 31], and is recommended by the International Workshop on CLL for use in clinical trials aimed at maximizing the depth of remission in patients with CLL [4]. However, several different methods of varying sensitivity are used to measure MRD, not all of which have been standardized, making comparability of test results between laboratories difficult [19, 20]. The vital role that MRD assays play in clinical decision-making necessitates not only assay standardization but also analytical validation, to enable a full understanding of the capability and limitations of each assay and to ensure that it is fit for the intended purpose of monitoring MRD.

We report the analytical validation of the clonoSEQ Assay in bone marrow samples from patients with ALL, CLL, and MM, tested across a range of DNA inputs suitable for clonotype detection and MRD monitoring. The clonoSEQ Assay has high sensitivity, with an LoD of 1.903 malignant cells and an LoQ of 2.390 malignant cells, across DNA input levels ranging from 200 ng to 40 μg, and it provides linear and accurate measurements over several orders of magnitude of MRD frequency. Like the LoD and LoQ, the precision of the clonoSEQ Assay is similar across disease indications. Since the key analytical features of the assay (LoD, LoQ, and precision) are a function of malignant cell abundance but not total DNA input, a given MRD frequency may have better performance characteristics at higher DNA inputs: for a fixed MRD threshold (e.g., 10^− 6^), sensitivity and quantitation will improve with more input DNA.

Clinical application of standardized diagnostic assays necessitates accuracy and reproducibility. Repeatability and reproducibility of the clonoSEQ Assay were nearly identical at each level of input cancer cells, showing that almost all observed variance was attributable to residual error and confirming that MRD frequencies measured by the assay are robust to different reagent lots, operators, instruments, and processing runs. Furthermore, since MRD assays may be used to inform treatment decisions, reliability is a key characteristic of any new diagnostic. Nucleotide sequence accuracy error rates of the clonoSEQ Assay were extremely low, indicating that sequence error constitutes a very small risk for generating false negative results. This may be of particular value in tracking minor clonotypes and monitoring clonal evolution.

One strength of the clonoSEQ Assay is its ability to track multiple receptor sequences from the same clonal population of malignant cells. This feature allows the assay to have an LoD below the theoretical Poisson limit of 3 malignant cells for a 95% detection rate. gDNA templates from each sequence are independently sampled into the assay, so even if one sequence from a low-level clone fails to be included into the gDNA pool, another sequence may still be sampled and detected. Tracking multiple sequences also improves the precision of the clonoSEQ Assay; Poisson sampling limits the precision of any particular sequence to ~ 70% CV near the LoD, and the assay is able to approach this level of precision by combining information across multiple tracked sequences per patient.

Non-uniqueness of receptor sequences can also be alleviated in this way; even if some rearrangements in a malignant clone are not complex enough to be completely absent from healthy clones, there is often another rearrangement that is highly unique to the malignancy. In rare cases where a patient’s cancer carries only rearrangements of intermediate uniqueness (i.e., sequences that have a low, but non-zero, probability of appearing in a healthy repertoire), MRD can still be tracked as long as care is taken to evaluate the possibility of false detection at low levels. The clonoSEQ Assay addresses such cases by using a large database of previously observed Ig rearrangements to assign a uniqueness score to each sequence, which represents its likelihood of being detected in a healthy repertoire. When a non-zero MRD result is driven by a sequence of intermediate uniqueness (as observed in a small fraction of measurements in the LoB study), this information is included in the report provided to clinicians and patients to inform decision-making.

The clonoSEQ Assay has some limitations. Unlike flow cytometry methods, but like other PCR based methods like ASO-PCR, the clonoSEQ Assay requires a pretreatment or diagnostic sample with relatively high disease burden to identify disease-associated clonotypes. While samples are usually available, the need for ID samples can limit access at times, and this study was not designed to evaluate the ability of the assay to identify disease clonotypes at tumor burdens near the threshold of detection in diagnostic samples. By comparison, the ability of flow cytometry to detect low levels of disease may depend on both the volume and cellularity of sampled input material, which may be a problem during treatment if bone marrow samples are aplastic [3]. Near the LoD, the clonoSEQ Assay has a slight upward bias that may cause MRD frequencies to be overestimated. In patients lacking a rearranged Ig locus (e.g., in a small subset of patients with B-cell precursor ALL whose transformed clone may be so immature that its immune receptor loci have not yet rearranged and are still in the germline configuration), other methods of monitoring MRD must be employed. Of the clinical samples used in these analytical validation studies, 4 out of the 115 (3.5%) samples that qualified for analysis had no detectable clonotype sequence, which is consistent with levels in published studies using the clonoSEQ Assay [32–37]. Finally, the clonoSEQ Assay is highly optimized for MRD detection and quantification; while it may lack the generality of some open-source platforms for processing B-cell repertoire data [38, 39], it achieves a high level of performance in MRD testing by tight integration between chemistry and software, and by providing built-in solutions for problems like non-unique sequences and accounting for multiple receptor sequences within the same malignant clone.

The clonoSEQ Assay detects disease below a threshold of 1 in 10^6^ of the total nucleated cell population, assuming a requisite number of total cells has been provided. Thus, provided the disease burden is at least 6.77 × 10^− 7^ (6.02 × 10^− 7^–7.61 × 10^− 7^) cells with 20 μg of input DNA, the clonoSEQ Assay detects disease 95% of the time.

## Conclusions

As demonstrated by the analytical validation data presented here, the clonoSEQ Assay is a robust, highly sensitive, and accurate method for quantifying and tracking MRD in bone marrow samples from patients with ALL or MM, or peripheral blood samples from patients with CLL.

## Supplementary information

**Additional file 1: Table S1.** Detailed list of clinical samples and cell lines selected for the analytical evaluation studies.

**Additional file 2: Figure S1.** Precision study PCR run execution map.

**Additional file 3: Figure S2.** Probit model plot to calculate the LoD.

**Additional file 4: Figure S3.** Verification of LoD (top) and LoQ (bottom) across the tested range of total input DNA.

**Additional file 5: Table S2.** Precision of the clonoSEQ Assay in MM samples.

**Additional file 6: Table S3.** Precision of the clonoSEQ Assay in ALL samples.

**Additional file 7: Table S4.** Precision of the clonoSEQ Assay in CLL samples.

**Additional file 8: Table S5.** Linearity of the clonoSEQ Assay using cell lines.

**Additional file 9: Table S6.** Linearity of the clonoSEQ Assay using clinical specimens from patients with ALL, CLL, and MM.

**Additional file 10: Figure S4.** Comparison of disease burden (percent of malignant cells out of total nucleated cells) estimated by the clonoSEQ Assay and mpFC in undiluted material from the 66 clinical samples and 9 cell lines used in this study.

**Additional file 11: Figure S5.** Bias estimates in quantitative clonoSEQ Assay MRD measurements in ALL, CLL, and MM.

## Data Availability

The datasets used and analyzed for this manuscript are available from the corresponding author on reasonable request.

## Abbreviations

%CV: Percentage coefficient of variation
ALL: Acute lymphocytic leukemia
ANOVA: Analysis of variance
ASO-PCR: Allele-specific oligonucleotide real-time quantitative polymerase chain reaction
ATCC: American Type Culture Collection
BCR: B-cell receptor
BMA: Bone marrow aspirate
CI: Confidence interval
CLL: Chronic lymphocytic leukemia
EFS: Event-free survival
gDNA: Genomic DNA
HR: Hazard ratio
ID: Diagnostic
Ig: Immunoglobulin
LoB: Limit of blank
LoD: Limit of detection
LoQ: Limit of quantitation
MM: Multiple myeloma
mpFC: Multiparameter flow cytometry
MRD: Minimal residual disease
NGS: Next generation sequencing
OPA: Overall percent agreement
OS: Overall survival
RMSE: Root-mean-square error
RFS: Relapse-free survival

## References

[1] Bassan R, Bruggemann M, Radcliffe HS, Hartfield E, Kreuzbauer G, Wetten S (2019). A systematic literature review and meta-analysis of minimal residual disease as a prognostic indicator in adult B-cell acute lymphoblastic leukemia. Haematologica.

[2] Berry DA, Zhou S, Higley H, Mukundan L, Fu S, Reaman GH (2017). Association of minimal residual disease with clinical outcome in pediatric and adult acute lymphoblastic leukemia: a meta-analysis. JAMA Oncol.

[3] Bruggemann M, Kotrova M (2017). Minimal residual disease in adult ALL: technical aspects and implications for correct clinical interpretation. Hematology Am Soc Hematol Educ Program.

[4] Hallek M, Cheson BD, Catovsky D, Caligaris-Cappio F, Dighiero G, Dohner H (2018). iwCLL guidelines for diagnosis, indications for treatment, response assessment, and supportive management of CLL. Blood.

[5] Health Quality O (2016). Minimal residual disease evaluation in childhood acute lymphoblastic leukemia: a clinical evidence review. Ont Health Technol Assess Ser.

[6] Landgren O, Rustad EH (2019). Meeting report: advances in minimal residual disease testing in multiple myeloma 2018. Adv Cell Gene Ther.

[7] Yanamandra U, Kumar SK (2018). Minimal residual disease analysis in myeloma - when, why and where. Leuk Lymphoma.

[8] Munshi NC, Avet-Loiseau H, Rawstron AC, Owen RG, Child JA, Thakurta A (2017). Association of minimal residual disease with superior survival outcomes in patients with multiple myeloma: a meta-analysis. JAMA Oncol.

[9] Molica S, Giannarelli D, Montserrat E (2019). Minimal residual disease and survival outcomes in patients with chronic lymphocytic leukemia: a systematic review and meta-analysis. Clin Lymphoma Myeloma Leuk.

[10] Caers J, Garderet L, Kortum KM, O'Dwyer ME, van de Donk N, Binder M (2018). European myeloma Network recommendations on tools for the diagnosis and monitoring of multiple myeloma: what to use and when. Haematologica.

[11] Eichhorst B, Robak T, Montserrat E, Ghia P, Hillmen P, Hallek M (2015). Chronic lymphocytic leukaemia: ESMO clinical practice guidelines for diagnosis, treatment and follow-up. Ann Oncol.

[12] Hoelzer D, Bassan R, Dombret H, Fielding A, Ribera JM, Buske C (2016). Acute lymphoblastic leukaemia in adult patients: ESMO clinical practice guidelines for diagnosis, treatment and follow-up. Ann Oncol.

[13] Kumar S, Paiva B, Anderson KC, Durie B, Landgren O, Moreau P (2016). International myeloma working group consensus criteria for response and minimal residual disease assessment in multiple myeloma. Lancet Oncol.

[14] Moreau P, San Miguel J, Sonneveld P, Mateos MV, Zamagni E, Avet-Loiseau H (2017). Multiple myeloma: ESMO Clinical Practice Guidelines for diagnosis, treatment and follow-up. Ann Oncol.

[15] National Comprehensive Cancer Network. Referenced with permission from the NCCN Clinical Practice Guidelines in Oncology (NCCN Guidelines®) for Acute Lymphoblastic Leukemia V.2.2019. © National Comprehensive Cancer Network, Inc. 2019. All rights reserved. Accessed 6 June 2019. To view the most recent and complete version of the guideline, go online to NCCN.org. 2019.

[16] National Comprehensive Cancer Network. Referenced with permission from the NCCN Clinical Practice Guidelines in Oncology (NCCN Guidelines®) for Chronic Lymphocytic Leukemia/Small Lymphocytic Leukemia V.5.2019. © National Comprehensive Cancer Network, Inc. 2019. All rights reserved. Accessed 6 June 2019. To view the most recent and complete version of the guideline, go online to NCCN.org. 2019.

[17] National Comprehensive Cancer Network. Referenced with permission from the NCCN Clinical Practice Guidelines in Oncology (NCCN Guidelines®) for Multiple Myeloma V.2.2019. © National Comprehensive Cancer Network, Inc. 2019. All rights reserved. Accessed 6 June 2019. To view the most recent and complete version of the guideline, go online to NCCN.org. 2019.

[18] National Comprehensive Cancer Network. Referenced with permission from the NCCN Clinical Practice Guidelines in Oncology (NCCN Guidelines®) for Pediatric Acute Lymphoblastic Leukemia V.1.2020. © National Comprehensive Cancer Network, Inc. 2019. All rights reserved. Accessed July 1, 2019. To view the most recent and complete version of the guideline, go online to NCCN.org. 2019.

[19] Dogliotti I, Drandi D, Genuardi E, Ferrero S. New molecular technologies for minimal residual disease evaluation in B-Cell lymphoid malignancies. J Clin Med. 2018;7(9):288. 10.3390/jcm7090288 PMC6162632 30231510

[20] Sanchez R, Ayala R, Martinez-Lopez J. Minimal residual disease monitoring with next-generation sequencing methodologies in hematological malignancies. Int J Mol Sci. 2019;20(11):2832. 10.3390/ijms20112832 PMC6600313 31185671

[21] Mateos MV, Dimopoulos MA, Cavo M, Suzuki K, Jakubowiak A, Knop S (2018). Daratumumab plus bortezomib, melphalan, and prednisone for untreated myeloma. N Engl J Med.

[22] Monter A, Nomdedeu JF (2019). ClonoSEQ assay for the detection of lymphoid malignancies. Expert Rev Mol Diagn.

[23] Tholen DW, Kroll M, Astles JR, Caffo AL, Happe TM, Krouwer J (2003). Evaluation of the linearity of quantitative measurement procedures: a statistical approach; approved guideline. Clin Lab Stand Inst.

[24] Pierson-Perry JF, Vaks JE, Durham AP, Fischer C, Gutenbrunner C, Hillyard D (2012). Evaluation of detection capability for clinical laboratory measurement procedures; approved guideline—second edition. Clin Lab Stand Inst.

[25] McEnroe RJ, Durham AP, Goldford MD, Kondratovich MV, Lababidi S, Magari R (2014). Evaluation of precision of quantitative measurement procedures; approved guideline - third edition. Clin Lab Stand Inst.

[26] Budd JR, Durham AP, Gwise TE, Iriarte B, Kallner A, Linnet K (2013). Measurement procedure comparison and bias estimation using patient samples: approved guideline - third edition. Clin Lab Stand Inst.

[27] Sadler WA (2008). Error models for immunoassays. Ann Clin Biochem.

[28] Krouwer JS, Schlain B (1993). A method to quantify deviations from assay linearity. Clin Chem.

[29] van Dongen JJ, van der Velden VH, Bruggemann M, Orfao A (2015). Minimal residual disease diagnostics in acute lymphoblastic leukemia: need for sensitive, fast, and standardized technologies. Blood.

[30] Paiva B, Vidriales MB, Cervero J, Mateo G, Perez JJ, Montalban MA (2008). Multiparameter flow cytometric remission is the most relevant prognostic factor for multiple myeloma patients who undergo autologous stem cell transplantation. Blood.

[31] Rawstron AC, Child JA, de Tute RM, Davies FE, Gregory WM, Bell SE (2013). Minimal residual disease assessed by multiparameter flow cytometry in multiple myeloma: impact on outcome in the Medical Research Council myeloma IX study. J Clin Oncol.

[32] Faham M, Zheng J, Moorhead M, Carlton VE, Stow P, Coustan-Smith E (2012). Deep-sequencing approach for minimal residual disease detection in acute lymphoblastic leukemia. Blood.

[33] Logan AC, Vashi N, Faham M, Carlton V, Kong K, Buno I (2014). Immunoglobulin and T cell receptor gene high-throughput sequencing quantifies minimal residual disease in acute lymphoblastic leukemia and predicts post-transplantation relapse and survival. Biol Blood Marrow Transplant.

[34] Mannis GN, Martin TG, Damon LE, Andreadis C, Olin RL, Kong KA (2016). Quantification of acute lymphoblastic leukemia clonotypes in leukapheresed peripheral blood progenitor cells predicts relapse risk after autologous hematopoietic stem cell transplantation. Biol Blood Marrow Transplant.

[35] Sala Torra O, Othus M, Williamson DW, Wood B, Kirsch I, Robins H (2017). Next-generation sequencing in adult B cell acute lymphoblastic leukemia patients. Biol Blood Marrow Transplant.

[36] Wood B, Wu D, Crossley B, Dai Y, Williamson D, Gawad C (2018). Measurable residual disease detection by high-throughput sequencing improves risk stratification for pediatric B-ALL. Blood.

[37] Wu D, Emerson RO, Sherwood A, Loh ML, Angiolillo A, Howie B (2014). Detection of minimal residual disease in B lymphoblastic leukemia by high-throughput sequencing of IGH. Clin Cancer Res.

[38] Cortina-Ceballos B, Godoy-Lozano E, Samano-Sanchez H, Aguilar-Salgado A, Velasco-Herrera M, Vargas-Vhavez C (2015). Reconstrructing and mining the B cell repertoire with ImmunediveRsity. MAbs.

[39] Lopez-Santibanez-Jacome L, Avendano-Vazquez E, Fabian C (2019). The pipeline repertorie for Ig-Seq analysis. Front Immunol.

