# Supplementary material for: Analytical evaluation of the clonoSEQ Assay for establishing measurable (minimal) residual disease in acute lymphoblastic leukemia, chronic lymphocytic leukemia, and multiple myeloma
Source: BMC Cancer. 2020 Jun 30;20:612. doi: 10.1186/s12885-020-07077-9 (PMC7325652; doi:10.1186/s12885-020-07077-9)
Supplement: Supplementary file 1 — Additional file 1: Table S1. Detailed list of clinical samples and cell lines selected for the analytical evaluation studies. [file 12885_2020_7077_MOESM1_ESM.docx]

Additional file 1

**Table S1** Detailed list of clinical samples and cell lines selected for the analytical evaluation studies

| Study sample ID | Disease type | Sample type | Calibrated receptor |
| --- | --- | --- | --- |
| 31113001003_ALL_BMA | ALL | BMA | IgH;IgL |
| 31125003003_ALL_BMA | ALL | BMA | IgH;IgL |
| 31125005003_ALL_BMA | ALL | BMA | IgH |
| 31127001004_ALL_BMA | ALL | BMA | IgHD |
| 31148005001_ALL_BMA | ALL | BMA | IgK;IgL |
| 31148005010_ALL_BMA | ALL | BMA | IgH;IgK |
| 31163005001_ALL_BMA | ALL | BMA | IgH;IgK;IgL |
| 31165007002_ALL_BMA | ALL | BMA | IgH;IgK |
| BCH001_ALL_BMMC | ALL | BMMC | IgH;IgK;IgL |
| BCH003_ALL_BMMC | ALL | BMMC | IgH;IgK;IgL |
| BCH004_ALL_BMMC | ALL | BMMC | IgH;IgK |
| BCH005_ALL_BMMC | ALL | BMMC | IgH;IgK |
| BCH006_ALL_BMMC | ALL | BMMC | IgH |
| BCH009_ALL_BMMC | ALL | BMMC | IgH |
| BCH012_ALL_BMMC | ALL | BMMC | IgH;IgK;IgHD;IgL |
| B-ALL_1_ALL_BMA | ALL | BMA | IgH;IgK;IgHD |
| B-ALL_10_ALL_BMA | ALL | BMA | IgH |
| B-ALL_2_ALL_BMA | ALL | PB | IgH;IgK;IgL |
| B-ALL_3_ALL_BMA | ALL | BMA | IgH;IgK |
| B-ALL_4_ALL_BMA | ALL | BMA | IgH;IgK |
| B-ALL_5_ALL_BMA | ALL | BMA | IgH;IgK |
| B-ALL_8_ALL_BMA | ALL | BMA | IgH;IgK;IgL |
| B-ALL_9_ALL_BMA | ALL | BMA | IgH;IgK |
| SUP-B15_cell_line^a^ | ALL | gDNA | IgK;IgH |
| GM-20390_cell_line^a^ | ALL | gDNA | IgK;IgH |
| GM14952_cell_line^a^,^b^ | ALL | gDNA | IgK;IgH;IgHD |
| CLL467BM_CLL_BMA | CLL | BMMC | IgH;IgK;IgHD;IgL |
| CLL468BM_CLL_BMA | CLL | BMMC | IgH;IgK;IgHD |
| CLL470BM_CLL_BMMC | CLL | BMMC | IgH;IgK;IgHD |
| CLL472BM_CLL_BMMC | CLL | BMMC | IgH;IgHD |
| CLL474BM_CLL_BMMC | CLL | BMMC | IgH |
| CLL475BM_CLL_BMMC | CLL | BMMC | IgK;IgH;IgHD |
| CLL476BM_CLL_BMMC | CLL | BMMC | IgH;IgHD |
| CLL477BM_CLL_BMMC | CLL | BMMC | IgK;IgH;IgHD |
| AS071-14_PBMC_CLL_PBMC | CLL | PBMC | IgH;IgK;IgHD |
| CA045-13_PBMC_CLL_PBMC | CLL | PBMC | IgH;IgK;IgHD |
| IN053-16_PBMC_CLL_PBMC | CLL | PBMC | IgH;IgHD;IgL |
| MS101-14_PBMC_CLL_PBMC | CLL | PBMC | IgH;IgK;IgL |
| PM013-15_PBMC_CLL_PBMC | CLL | PBMC | IgH;IgK;IgHD |
| PU066-15_PBMC_CLL_PBMC | CLL | PBMC | IgH;IgK;IgHD;IgL |
| RC123-13_PBMC_CLL_PBMC | CLL | PBMC | IgH;IgK |
| RM006-13_PBMC_CLL_PBMC | CLL | PBMC | IgH;IgK |
| SM009-13_PBMC_CLL_PBMC | CLL | PBMC | IgH;IgK |
| VR060-13_PBMC_CLL_PBMC | CLL | PBMC | IgH;IgK;IgHD |
| CLL_1_CLL_Blood | CLL | PB | IgH;IgHD |
| CLL_2_CLL_Blood | CLL | PB | IgH;IgK;IgHD;IgL |
| CLL_3_CLL_Blood | CLL | PB | IgH;IgK;IgHD;IgL |
| CLL_6_CLL_Blood | CLL | PB | IgH;IgK;IgL |
| CLL_8_CLL_Blood | CLL | BMA | IgH;IgK;IgHD;IgL |
| PGA-1_cell_line^a^ | CLL | gDNA | IgK;IgH;IgHD;IgL |
| HG-3_cell_line^a^ | CLL | gDNA | IgH;IgHD;IgL |
| MEC-1_cell_line^a^,^b^ | CLL | gDNA | IgK;IgH;IgHD |
| 230822_MM_PlasmaCell | MM | BMMC | IgH;IgHD;IgL |
| MYELOMA_1_MM_BMA | MM | BMA | IgK;IgL |
| UPN56009_MM_BMMC | MM | BMMC | IgH;IgK;IgL |
| UPN68043_MM_BMMC | MM | BMMC | IgH;IgH;IgHD |
| UPN95122_MM_BMMC | MM | BMMC | IgH;IgK |
| CD138+_T11664_MM_CD138+ | MM | CD138+ cell pellet | IgK;IgH;IgL |
| CD138+_T11729_MM_CD138+ | MM | CD138+ cell pellet | IgK;IgH;IgHD |
| CD138+_T11805_MM_CD138+ | MM | CD138+ cell pellet | IgK;IgH;IgHD;IgL |
| MM435BM_MM_BMMC | MM | BMMC | IgH;IgK |
| UPN13284_MM_BMMC | MM | BMMC | IgH;IgK |
| UPN14460_MM_BMMC | MM | BMMC | IgH;IgK;IgHD |
| CD138+_T10673_MM_CD138+ | MM | CD138+ cell pellet | IgK;IgH |
| CD138+_T10729_MM_CD138+ | MM | CD138+ cell pellet | IgK;IgH;IgHD |
| CD138+_T10736_MM_CD138+ | MM | CD138+ cell pellet | IgK;IgH |
| CD138+_T10744_MM_CD138+ | MM | CD138+ cell pellet | IgK;IgH |
| CD138+_T10751_MM_CD138+ | MM | CD138+ cell pellet | IgK;IgHD |
| CD138+_T10764_MM_CD138+ | MM | CD138+ cell pellet | IgK;IgH;IgHD |
| CD138+_T10777_MM_CD138+ | MM | CD138+ cell pellet | IgK;IgH |
| CD138+_T10807_MM_CD138+ | MM | CD138+ cell pellet | IgK |
| CD138+_T11658_MM_CD138+ | MM | CD138+ cell pellet | IgH |
| CD138+_T11666_MM_CD138+ | MM | CD138+ cell pellet | IgH;IgHD |
| CD138+_T11685_MM_CD138+ | MM | CD138+ | IgK;IgH |
| CD138+_T11718_MM_CD138+ | MM | CD138+ cell pellet | IgH;IgHD;IgL |
| CD138+_T11746_MM_CD138+ | MM | CD138+ cell pellet | IgK;IgHD |
| CD138+_T11763_MM_CD138+ | MM | CD138+ cell pellet | IgK;IgH;IgHD |
| CD138+_T11820_MM_CD138+ | MM | CD138+ cell pellet | IgK;IgH |
| IM-9_cell_line^a^,^b^ | MM | gDNA | IgK;IgH;IgHD |
| U-266_cell_line^a^ | MM | gDNA | IgK;IgH;IgL |
| L-363_cell_line^a^ | MM | gDNA | IgK;IgL |

*ALL* acute lymphoblastic leukemia, *BMA* bone marrow aspirate, *BMMC* bone marrow-derived mononuclear cell, *CLL* chronic lymphocytic leukemia, *gDNA* genomic DNA, *MM* multiple myeloma, *PB* peripheral blood, *PBMC* peripheral blood mononuclear cells.

^a^Cell line gDNA used in the precision analysis.

^b^Cell line gDNA used in the LOB LOD and LOQ analyses.

Samples were identified based on (1) disease load > 30% by an orthogonal technology; (2) having calibrated clone(s); (3) having sequence independence, and (4) having sufficient material available to execute the AV studies.

Of the 72 clinical samples chosen, 6 had disease loads < 10% by clonoSEQ (3 MM, 1 CLL, 2 ALL) and were subsequently eliminated from all evaluation analyses.

Most of these samples included a mixture of tumor and non-tumor cells, but those with sample type “CD138+ cell pellet” were enriched for tumor cells by flow sorting to facilitate the identification of disease clones.
