# Supplementary material for: Analytical evaluation of the clonoSEQ Assay for establishing measurable (minimal) residual disease in acute lymphoblastic leukemia, chronic lymphocytic leukemia, and multiple myeloma
Source: BMC Cancer. 2020 Jun 30;20:612. doi: 10.1186/s12885-020-07077-9 (PMC7325652; doi:10.1186/s12885-020-07077-9)

Additional file 2

**Figure S1** Precision study PCR run execution map

NextSeq # = Number of particular sequencing machine. Different machines were used to ensure reproducibility of results.


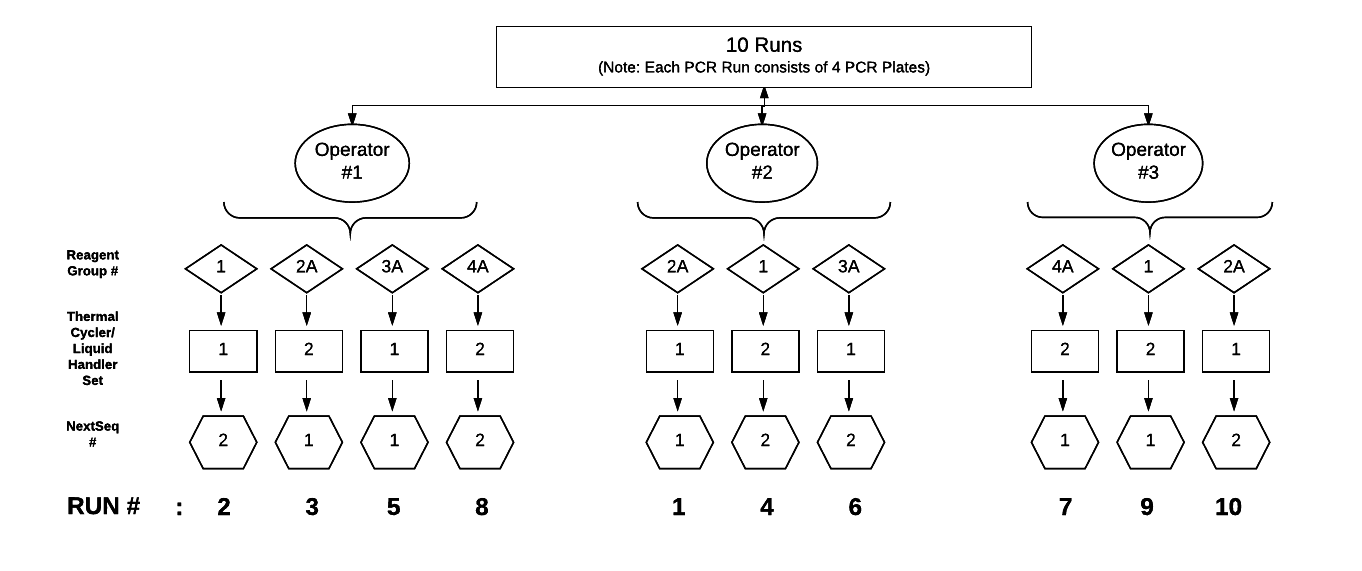

Supplement: Supplementary file 2 — Additional file 2: Figure S1. Precision study PCR run execution map. [file 12885_2020_7077_MOESM2_ESM.docx]
