# Supplementary material for: Analytical evaluation of the clonoSEQ Assay for establishing measurable (minimal) residual disease in acute lymphoblastic leukemia, chronic lymphocytic leukemia, and multiple myeloma
Source: BMC Cancer. 2020 Jun 30;20:612. doi: 10.1186/s12885-020-07077-9 (PMC7325652; doi:10.1186/s12885-020-07077-9)
Supplement: Supplementary file 3 — Additional file 3: Figure S2. Probit model plot to calculate the LoD. [file 12885_2020_7077_MOESM3_ESM.docx]

Additional file 3

**Figure S2** Probit model plot to calculate the LoD


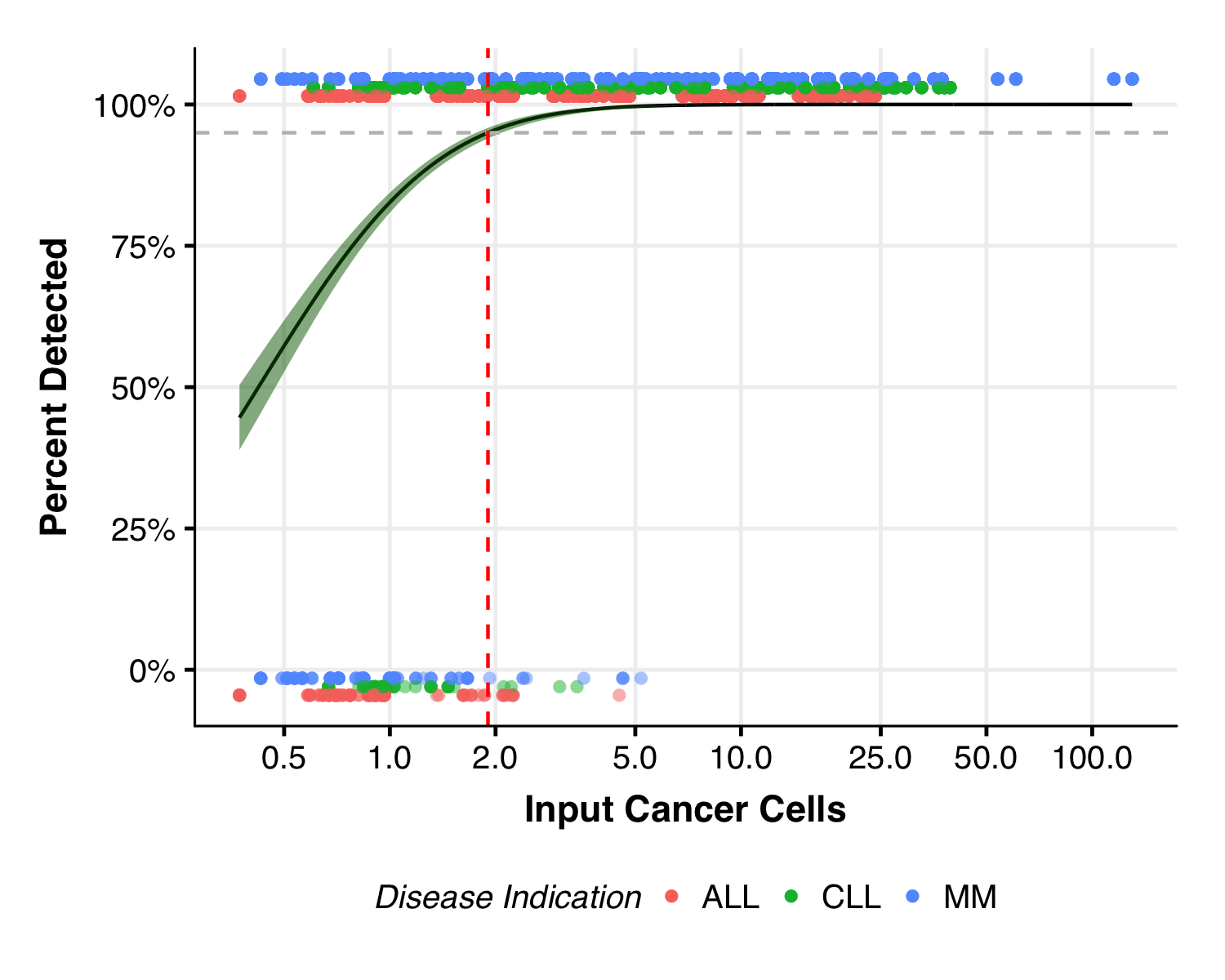
Red dashed line indicates LOD value. Gray dashed line indicates 95% detection threshold.

*ALL* acute lymphoblastic leukemia, *CLL* chronic lymphocytic leukemia, *LOD* limit of detection,
*MM* multiple myeloma.
