# Supplementary material for: Analytical evaluation of the clonoSEQ Assay for establishing measurable (minimal) residual disease in acute lymphoblastic leukemia, chronic lymphocytic leukemia, and multiple myeloma
Source: BMC Cancer. 2020 Jun 30;20:612. doi: 10.1186/s12885-020-07077-9 (PMC7325652; doi:10.1186/s12885-020-07077-9)
Supplement: Supplementary file 4 — Additional file 4: Figure S3. Verification of LoD (top) and LoQ (bottom) across the tested range of total input DNA. [file 12885_2020_7077_MOESM4_ESM.docx]

Additional file 4

**Figure S3** Verification of LoD (top) and LoQ (bottom) across the tested range of total input DNA
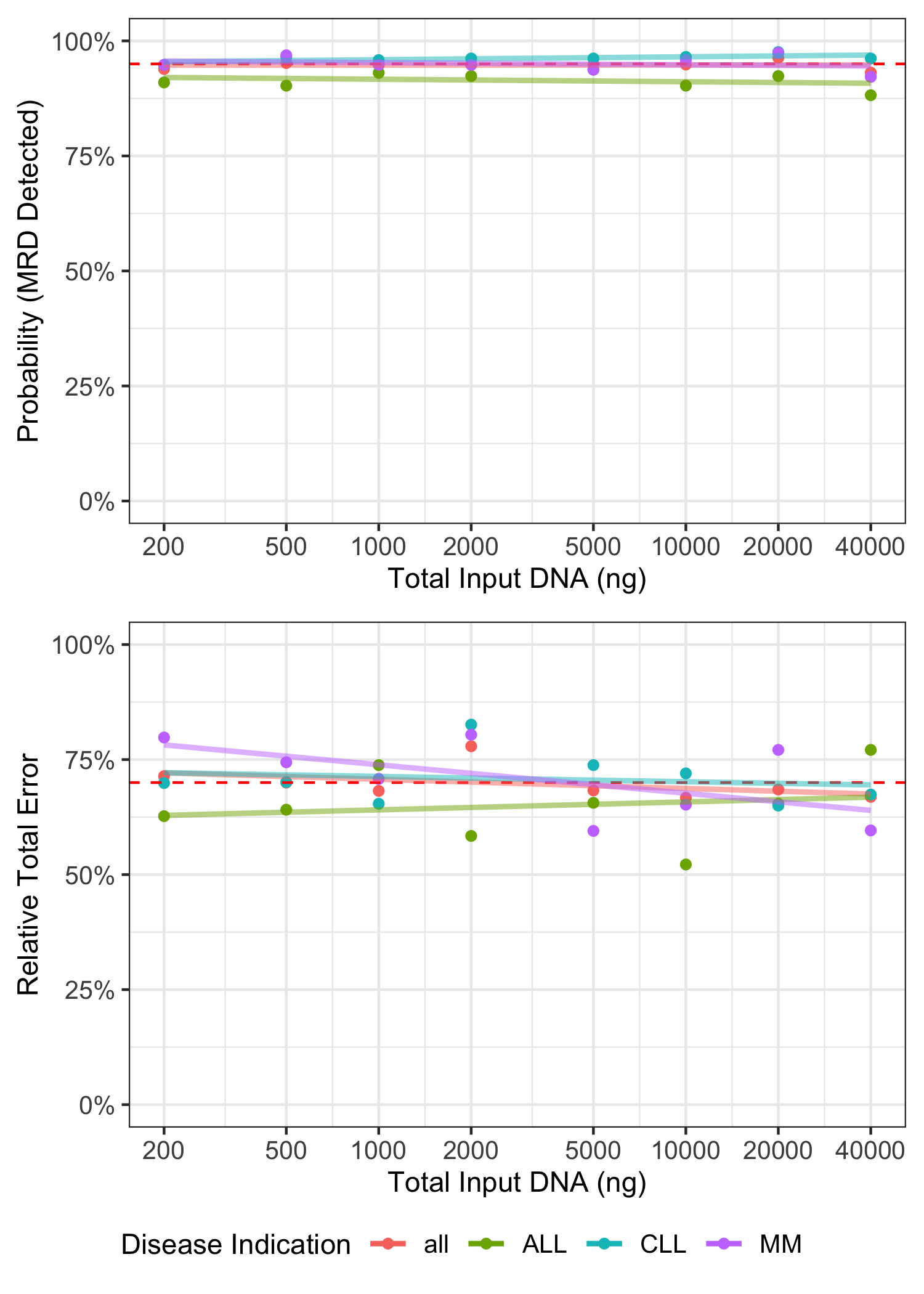


Red dashed lines are at 95% probability (LOD; top figure) and 70% relative total error
(LOQ; bottom figure). *ALL* acute lymphoblastic leukemia, *CLL* chronic lymphocytic leukemia, *LOD* limit of detection, *LOQ* limit of quantitation, *MM* multiple myeloma, *MRD* minimal residual disease.
