# Supplementary material for: Analytical evaluation of the clonoSEQ Assay for establishing measurable (minimal) residual disease in acute lymphoblastic leukemia, chronic lymphocytic leukemia, and multiple myeloma
Source: BMC Cancer. 2020 Jun 30;20:612. doi: 10.1186/s12885-020-07077-9 (PMC7325652; doi:10.1186/s12885-020-07077-9)
Supplement: Supplementary file 5 — Additional file 5: Table S2. Precision of the clonoSEQ Assay in MM samples. [file 12885_2020_7077_MOESM5_ESM.docx]

Additional file 5

**Table S2** Precision of the clonoSEQ Assay in MM Samples

| DNA Input | MRD Frequency | Measurements | Patients^a^ | %CV | Frequency Range  (95% CI) |
| --- | --- | --- | --- | --- | --- |
| 500 ng | 3.5x10^-5^ | 378 | 21 | 70.0 | 0–9.0x10^-5^ |
|  | 8.9x10^-5^ | 378 | 21 | 52.6 | 1.9x10^-5^–2.0x10^-4^ |
|  | 3.0x10^-4^ | 414 | 23 | 37.6 | 1.2x10^-4^–5.7x10^-4^ |
|  | 8.4x10^-4^ | 460 | 23 | 34.8 | 3.9x10^-4^–1.6x10^-3^ |
|  | 2.7x10^-3^ | 460 | 23 | 32.3 | 1.4x10^-3^–5.1x10^-3^ |
|  | 6.5x10^-3^ | 460 | 23 | 30.1 | 3.6x10^-3^–1.2x10^-2^ |
| 2 μg | 8.1x10^-6^ | 378 | 21 | 69.2 | 0–2.1x10^-5^ |
|  | 2.3x10^-5^ | 378 | 21 | 52.1 | 5.7x10^-6^–5.4x10^-5^ |
|  | 7.8x10^-5^ | 414 | 23 | 38.8 | 3.1x10^-5^–1.5x10^-4^ |
|  | 2.1x10^-4^ | 460 | 23 | 34.4 | 1.0x10^-4^–4.0x10^-4^ |
|  | 7.4x 0^-4^ | 460 | 23 | 32.4 | 3.7x10^-4^–1.4x10^-3^ |
|  | 2.0x10^-3^ | 460 | 23 | 30.2 | 1.1x10^-3^–3.8x10^-3^ |
| 20 μg | 8.6x10^-7^ | 378 | 21 | 65.6 | 0–2.1x10^-6^ |
|  | 2.3x10^-6^ | 378 | 21 | 51.7 | 5.1x10^-7^–5.0x10^-6^ |
|  | 8.2x10^-6^ | 414 | 23 | 37.2 | 3.5x10^-6^–1.5x10^-5^ |
|  | 2.3x10^-5^ | 460 | 23 | 33.3 | 1.2x10^-5^–4.5x10^-5^ |
|  | 7.6x10^-5^ | 460 | 23 | 31.1 | 4.1x10^-5^–1.4x10^-4^ |
|  | 2.1x10^-4^ | 414 | 23 | 29.5 | 1.2x10^-4^–3.7x10^-4^ |

*%CV* percentage coefficient of variation, *CI* confidence interval, *MM* multiple myeloma, *MRD* minimal residual disease.

^a^Some contrived samples included a subset of patient samples.
