# Supplementary material for: Analytical evaluation of the clonoSEQ Assay for establishing measurable (minimal) residual disease in acute lymphoblastic leukemia, chronic lymphocytic leukemia, and multiple myeloma
Source: BMC Cancer. 2020 Jun 30;20:612. doi: 10.1186/s12885-020-07077-9 (PMC7325652; doi:10.1186/s12885-020-07077-9)
Supplement: Supplementary file 6 — Additional file 6: Table S3. Precision of the clonoSEQ Assay in ALL samples. [file 12885_2020_7077_MOESM6_ESM.docx]

Additional file 6

**Table S3** Precision of the clonoSEQ Assay in ALL Samples

| DNA Input | MRD Frequency | Measurements | Patients | %CV | Frequency range  (95% CI) |
| --- | --- | --- | --- | --- | --- |
| 500 ng | 3.2x10^-5^ | 378 | 21 | 76.9 | 0–9.3x10^-5^ |
|  | 8.6x10^-5^ | 378 | 21 | 54.1 | 1.0x10^-5^–1.9x10^-4^ |
|  | 2.8x10^-4^ | 378 | 21 | 33.0 | 1.0x10^-4^–4.8x10^-4^ |
|  | 7.6x10^-4^ | 420 | 21 | 29.3 | 3.4x10^-4^–1.2x10^-3^ |
|  | 2.5x10^-3^ | 420 | 21 | 26.5 | 1.0x10^-3^–4.0x10^-3^ |
|  | 6.0x10^-3^ | 420 | 21 | 25.9 | 2.2x10^-3^–9.8x10^-3^ |
| 2 μg | 7.6x10^-6^ | 378 | 21 | 74.1 | 0–2.0x10^-5^ |
|  | 2.1x10^-5^ | 378 | 21 | 47.4 | 3.8x10^-6^–4.0x10^-5^ |
|  | 6.8x10^-5^ | 378 | 21 | 33.3 | 2.2x10^-5^–1.1x10^-4^ |
|  | 1.9x10^-4^ | 420 | 21 | 29.1 | 7.0x10^-5^–3.1x10^-4^ |
|  | 6.8x10^-4^ | 420 | 21 | 27.1 | 2.5x10^-4^–1.1x10^-3^ |
|  | 1.9x10^-3^ | 420 | 21 | 25.9 | 6.9x10^-4^–3.1x10^-3^ |
| 20 μg | 8.5x10^-7^ | 378 | 21 | 74.8 | 0–2.2x10^-6^ |
|  | 2.3x10^-6^ | 378 | 21 | 49.8 | 4.0x10^-7^–4.7x10^-6^ |
|  | 7.5x10^-6^ | 378 | 21 | 34.5 | 2.6x10^-6^–1.3x10^-5^ |
|  | 2.1x10^-5^ | 420 | 21 | 27.9 | 8.5x10^-6^–3.4x10^-5^ |
|  | 6.9x10^-5^ | 420 | 21 | 26.7 | 2.5x10^-5^–1.1 x 10^-4^ |
|  | 2.0x10^-4^ | 378 | 21 | 26.5 | 7.1x10^-5^–3.3x10^-4^ |

*%CV* percentage coefficient of variation, *ALL* acute lymphoblastic leukemia, *CI* confidence interval,

*MRD* minimal residual disease.
