# Supplementary material for: Analytical evaluation of the clonoSEQ Assay for establishing measurable (minimal) residual disease in acute lymphoblastic leukemia, chronic lymphocytic leukemia, and multiple myeloma
Source: BMC Cancer. 2020 Jun 30;20:612. doi: 10.1186/s12885-020-07077-9 (PMC7325652; doi:10.1186/s12885-020-07077-9)
Supplement: Supplementary file 7 — Additional file 7: Table S4. Precision of the clonoSEQ Assay in CLL samples. [file 12885_2020_7077_MOESM7_ESM.docx]

Additional file 7

**Table S4** Precision of the clonoSEQ Assay in CLL Samples

| DNA Input | MRD Frequency | Measurements | Patients | %CV | Frequency range  (95% CI) |
| --- | --- | --- | --- | --- | --- |
| 500 ng | 4.4x10^-5^ | 396 | 22 | 57.7 | 0–1.0x10^-4^ |
|  | 1.0x10^-4^ | 396 | 22 | 42.6 | 3.4x10^-5^–2.0x10^-4^ |
|  | 3.4x10^-4^ | 396 | 22 | 27.8 | 1.7x10^-4^–5.6x10^-4^ |
|  | 9.2x10^-4^ | 440 | 22 | 24.2 | 5.6x10^-4^–1.5x10^-3^ |
|  | 2.9x10^-3^ | 440 | 22 | 21.2 | 1.9x10^-3^–4.4x10^-3^ |
|  | 7.1x10^-3^ | 440 | 22 | 19.6 | 4.5x10^-3^–1.0x10^-2^ |
| 2 ug | 1.0x10^-5^ | 396 | 22 | 60.1 | 0–2.4x10^-5^ |
|  | 2.6x10^-5^ | 396 | 22 | 44.5 | 9.3x10^-6^–5.4x10^-5^ |
|  | 8.5x10^-5^ | 396 | 22 | 28.9 | 4.5x10^-5^–1.4x10^-4^ |
|  | 2.3x10^-4^ | 440 | 22 | 23.5 | 1.4x10^-4^–3.8x10^-4^ |
|  | 8.2x10^-4^ | 440 | 22 | 21.4 | 5.1x10^-4^–1.3x10^-3^ |
|  | 2.2x10^-3^ | 440 | 22 | 19.2 | 1.4x10^-3^–3.2x10^-3^ |
| 20 ug | 1.1x10^-6^ | 396 | 22 | 59.8 | 0–2.6x10^-6^ |
|  | 2.9x10^-6^ | 396 | 22 | 40.7 | 1.0x10^-6^–5.6x10^-6^ |
|  | 9.1x10^-6^ | 396 | 22 | 27.2 | 5.4x10^-6^–1.5x10^-5^ |
|  | 2.6x10^-5^ | 440 | 22 | 22.1 | 1.6x10^-5^–3.9x10^-5^ |
|  | 8.4x10^-5^ | 440 | 22 | 19.9 | 5.2x10^-5^–1.2x10^-4^ |
|  | 2.3x10^-4^ | 396 | 22 | 18.5 | 1.6x10^-4^–3.3x10^-4^ |

*%CV* percentage coefficient of variation, *CI* confidence interval, *CLL* chronic lymphocytic leukemia, *MRD* minimal residual disease.
