# Supplementary material for: Analytical evaluation of the clonoSEQ Assay for establishing measurable (minimal) residual disease in acute lymphoblastic leukemia, chronic lymphocytic leukemia, and multiple myeloma
Source: BMC Cancer. 2020 Jun 30;20:612. doi: 10.1186/s12885-020-07077-9 (PMC7325652; doi:10.1186/s12885-020-07077-9)
Supplement: Supplementary file 8 — Additional file 8: Table S5. Linearity of the clonoSEQ Assay using cell lines. [file 12885_2020_7077_MOESM8_ESM.docx]

Additional file 8

**Table S5** Linearity of the clonoSEQ Assay using cell lines

|  |  |  | Combined analysis | | | Summary of individual patient analyses | |
| --- | --- | --- | --- | --- | --- | --- | --- |
| Disease indication | Input DNA | Tested range | Linear range (MRD frequency) | Slope | Intercept | Slope range | Intercept range |
| ALL | 200 ng | 0 to 1 | 0 to 1 | 1.015 | –0.109 | 0.988 to 1.047 | –0.313 to 0.016 |
|  | 2 μg | 0 to 1 | 3.0x10^-5^ to 0.3 | 0.988 | –0.172 | 0.976 to 0.996 | –0.391 to –0.042 |
|  | 20 μg | 0 to 0.1 | 0 to 0.1 | 0.952 | –0.262 | 0.882 to 0.991 | –0.628 to –0.051 |
|  | 40 μg | 0 to 0.1 | 0 to 0.1 | 0.99 | 0.080 | 0.981 to 0.991 | 0.062 to 0.102 |
| MM | 200 ng | 0 to 1 | 0 to 1 | 1.028 | –0.066 | 0.978 to 1.113 | –0.077 to –0.044 |
|  | 2 μg | 0 to 1 | 9.8x10^-6^ to 0.3 | 1.015 | –0.033 | 0.985 to 1.064 | –0.084 to 0.016 |
|  | 20 μg | 0 to 0.1 | 0 to 0.1 | 0.978 | –0.145 | 0.960 to 0.993 | –0.222 to –0.041 |
|  | 40 μg | 0 to 0.1 | 0 to 0.1 | 1.01 | 0.109 | 0.981 to 1.056 | 0.079 to 0.153 |
| CLL | 200 ng | 0 to 1 | 0 to 1 | 0.994 | 0.018 | 0.978 to 1.011 | –0.045 to 0.129 |
|  | 2 μg | 0 to 1 | 0 to 0.1 | 1.004 | 0.034 | 0.998 to 1.016 | –0.045 to 0.161 |
|  | 20 μg | 0 to 0.1 | 0 to 1 | 0.994 | –0.033 | 0.974 to 1.019 | –0.159 to 0.111 |

*ALL* acute lymphoblastic leukemia, *CLL* chronic lymphocytic leukemia, *MM* multiple myeloma, *MRD* minimal residual disease.
