# Supplementary material for: Analytical evaluation of the clonoSEQ Assay for establishing measurable (minimal) residual disease in acute lymphoblastic leukemia, chronic lymphocytic leukemia, and multiple myeloma
Source: BMC Cancer. 2020 Jun 30;20:612. doi: 10.1186/s12885-020-07077-9 (PMC7325652; doi:10.1186/s12885-020-07077-9)
Supplement: Supplementary file 9 — Additional file 9: Table S6. Linearity of the clonoSEQ Assay using clinical specimens from patients with ALL, CLL, and MM. [file 12885_2020_7077_MOESM9_ESM.docx]

Additional file 9

**Table S6** Linearity of the clonoSEQ Assay using clinical specimens from patients with ALL, CLL, and MM

|  |  |  | Combined analysis | | | Summary of individual patient analyses | |
| --- | --- | --- | --- | --- | --- | --- | --- |
| Disease indication | Input DNA | Tested range | Linear range  (MRD frequency) | Slope | Intercept | Slope range | Intercept range |
| ALL | 500 ng | 2.8x10^-5^ to 8.0x10^-3^ | 2.8x10^-5^ to 8.0x10^-3^ | 0.948 | –0.214 | 0.853 to 1.073 | –0.461 to 0.018 |
|  | 2 μg | 7.0x10^-6^ to 2.0x10^-3^ | 7.0x10 ^-6^ to 2.0x10^-3^ | 0.985 | –0.074 | 0.909 to 1.076 | –0.757 to 0.233 |
|  | 20 μg | 7.0x10^-7^ to 2.0x10^-4^ | 7.0x10^-7^ to 2.0x10^-4^ | 0.978 | –0.101 | 0.859 to 1.029 | –1.018 to 0.336 |
| MM | 500 ng | 2.8x10^-5^ to 8.0x10^-3^ | 2.8x10^-5^ to 8.0x10^-3^ | 0.962 | –0.143 | 0.853 to 1.148 | –0.462 to 0.183 |
|  | 2 μg | 7.0x10^-6^ to 2.0x10^-3^ | 7.0x10 ^-6^ to 2.0x10^-3^ | 0.986 | –0.04 | 0.924 to 1.068 | –0.341 to 0.246 |
|  | 20 μg | 7.0x10^-7^ to 2.0x10^-4^ | 7.0x10^-7^ to 2.0x10^-4^ | 0.985 | –0.034 | 0.933 to 1.075 | –0.419 to 0.611 |
| CLL | 500 ng | 2.8x10^-5^ to 8.0x10^-3^ | 2.8x10^-5^ to 8.0x10^-3^ | 0.916 | –0.216 | 0.847 to 1.004 | –0.450 to 0.011 |
|  | 2 μg | 7.0x10^-6^ to 2.0x10^-3^ | 7.0x10 ^-6^ to 2.0x10^-3^ | 0.964 | –0.057 | 0.877 to 1.043 | –0.358 to 0.248 |
|  | 20 μg | 7.0x10^-7^ to 2.0x10^-4^ | 7.0x10^-7^ to 2.0x10^-4^ | 0.984 | 0.013 | 0.924 to 1.048 | –0.419 to 0.272 |

*ALL* acute lymphoblastic leukemia, *CLL* chronic lymphocytic leukemia, *MM* multiple myeloma, *MRD* minimal residual disease.
