# Supplementary material for: Analytical evaluation of the clonoSEQ Assay for establishing measurable (minimal) residual disease in acute lymphoblastic leukemia, chronic lymphocytic leukemia, and multiple myeloma
Source: BMC Cancer. 2020 Jun 30;20:612. doi: 10.1186/s12885-020-07077-9 (PMC7325652; doi:10.1186/s12885-020-07077-9)
Supplement: Supplementary file 10 — Additional file 10: Figure S4. Comparison of disease burden (percent of malignant cells out of total nucleated cells) estimated by the clonoSEQ Assay and mpFC in undiluted material from the 66 clinical samples and 9 cell lines used in this study. [file 12885_2020_7077_MOESM10_ESM.docx]

Additional file 10

**Figure S4** Comparison of disease burden (percent of malignant cells out of total nucleated cells) estimated by the clonoSEQ Assay and mpFC in undiluted material from the 66 clinical samples and 9 cell lines used in this study.
