# Supplementary material for: Analytical evaluation of the clonoSEQ Assay for establishing measurable (minimal) residual disease in acute lymphoblastic leukemia, chronic lymphocytic leukemia, and multiple myeloma
Source: BMC Cancer. 2020 Jun 30;20:612. doi: 10.1186/s12885-020-07077-9 (PMC7325652; doi:10.1186/s12885-020-07077-9)
Supplement: Supplementary file 11 — Additional file 11: Figure S5. Bias estimates in quantitative clonoSEQ Assay MRD measurements in ALL, CLL, and MM. [file 12885_2020_7077_MOESM11_ESM.docx]

Additional file 11

**Figure S5** Bias estimates in quantitative clonoSEQ Assay MRD measurements in ALL, CLL, and MM

**
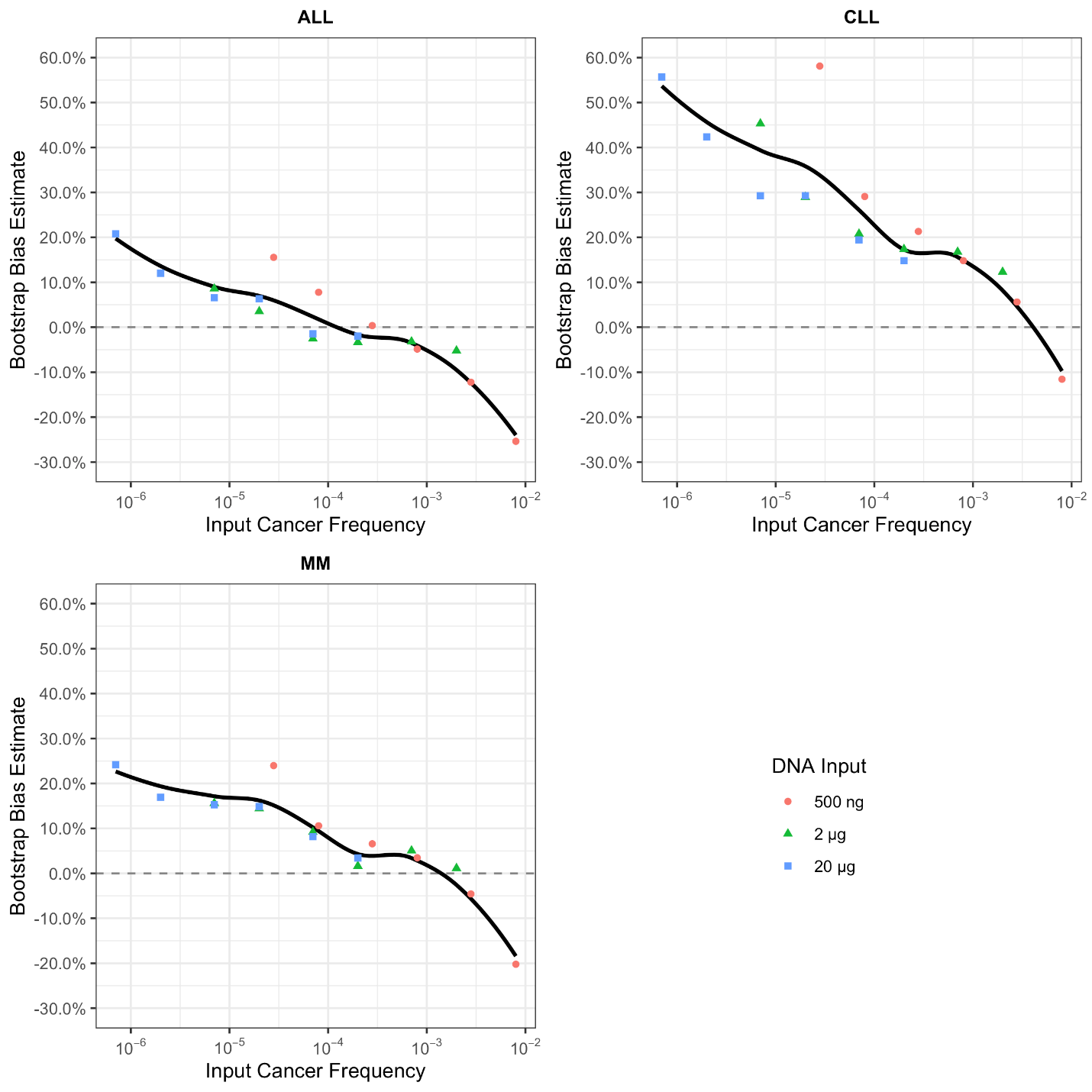
**

*ALL* acute lymphoblastic leukemia, *CLL* chronic lymphocytic leukemia, *MM* multiple myeloma.
